# Disruption of HSPA8-GEMIN5 interaction suppresses colorectal cancer by impaired splicing-translation coupling-mediated proteostasis imbalance

**DOI:** 10.1186/s13046-026-03645-2

**Published:** 2026-01-16

**Authors:** Fei Wang, Huiming Huang, Ruoxin Zhang, Xuejiao Wei, Zhuguo Wang, Xinyu Qiu, Yufeng Gao, Xiaoxue Wang, Wanying Xie, Hongbing Zhang, Pengfei Tu, Zhongdong Hu

**Affiliations:** 1 School of Chinese Materia Medica, Beijing University of Chinese Medicine, Beijing, 100029 China; 2 Modern Research Center for Traditional Chinese Medicine, Beijing Research Institute of Chinese Medicine, Beijing University of Chinese Medicine, Beijing, 100029 China; 3 Department of Physiology, State Key Laboratory of Common Mechanism Research for Major Diseases, Haihe Laboratory of Cell Ecosystem, Institute of Basic Medical Sciences and School of Basic Medicine, Chinese Academy of Medical Sciences and Peking Union Medical College, Beijing, 100005 China

**Keywords:** DSHK, Colorectal cancer, HSPA8, GEMIN5, Alternative splicing, Proteostasis

## Abstract

**Background:**

Colorectal cancer (CRC) is one of the most prevalent malignant tumors globally, and there is an urgent need for effective treatment strategies. The natural compound Deoxyshikonin (DSHK) has shown promising anti-tumor potential. However, the anti-CRC effects of DSHK and its molecular target remain unclear.

**Methods:**

The anti-CRC efficacy of DSHK was evaluated using human CRC cell lines, patient-derived organoids (PDOs), cell line-derived xenograft (CDX), and patient-derived organoid xenograft (PDOX) models. Target identification involved chemical proteomics, CETSA, SPR, and molecular dynamics simulations. Protein interactions were probed using SPIDER proximity labeling, Co-IP, GST pull-down, and confocal microscopy. The spatial distribution of interacting proteins was examined through high-density tissue microarrays, and functional pathways were explored via whole-transcriptome sequencing, rMATS, and ultrastructural imaging.

**Results:**

DSHK demonstrated potent antitumor efficacy in preclinical models of CRC. HSPA8 was identified as the direct molecular target of DSHK. Moreover, the anti-CRC effect of DSHK depended on HSPA8. Additionally, GEMIN5 was identified as a novel functional interactor of HSPA8 in CRC pathogenesis. The expression levels and co-localization intensity of HSPA8 and GEMIN5 were significantly higher in CRC tissues compared to adjacent normal tissues. Moreover, DSHK destabilized the oncogenic HSPA8-GEMIN5 complex, thereby triggering aberrant splicing of ribosomal protein-coding genes mediated by GEMIN5, thereby impeding functional ribosome biogenesis. Concomitantly, DSHK impaired HSPA8-mediated initiation factors interaction and destabilized the eIF4F complex, resulting in dysfunctional translation initiation.

**Conclusions:**

DSHK targeted the HSPA8-GEMIN5 interaction interface to impair ribosome biogenesis and dysregulating translation initiation to suppress protein synthesis. This study established the newly identified HSPA8-GEMIN5 complex as a molecular hub mediating “splicing-translation coupling” in CRC and provided a novel “dual-pathway intervention targeting splicing and translation” strategy inducing proteostasis imbalance for CRC therapy.

**Graphical Abstract:**

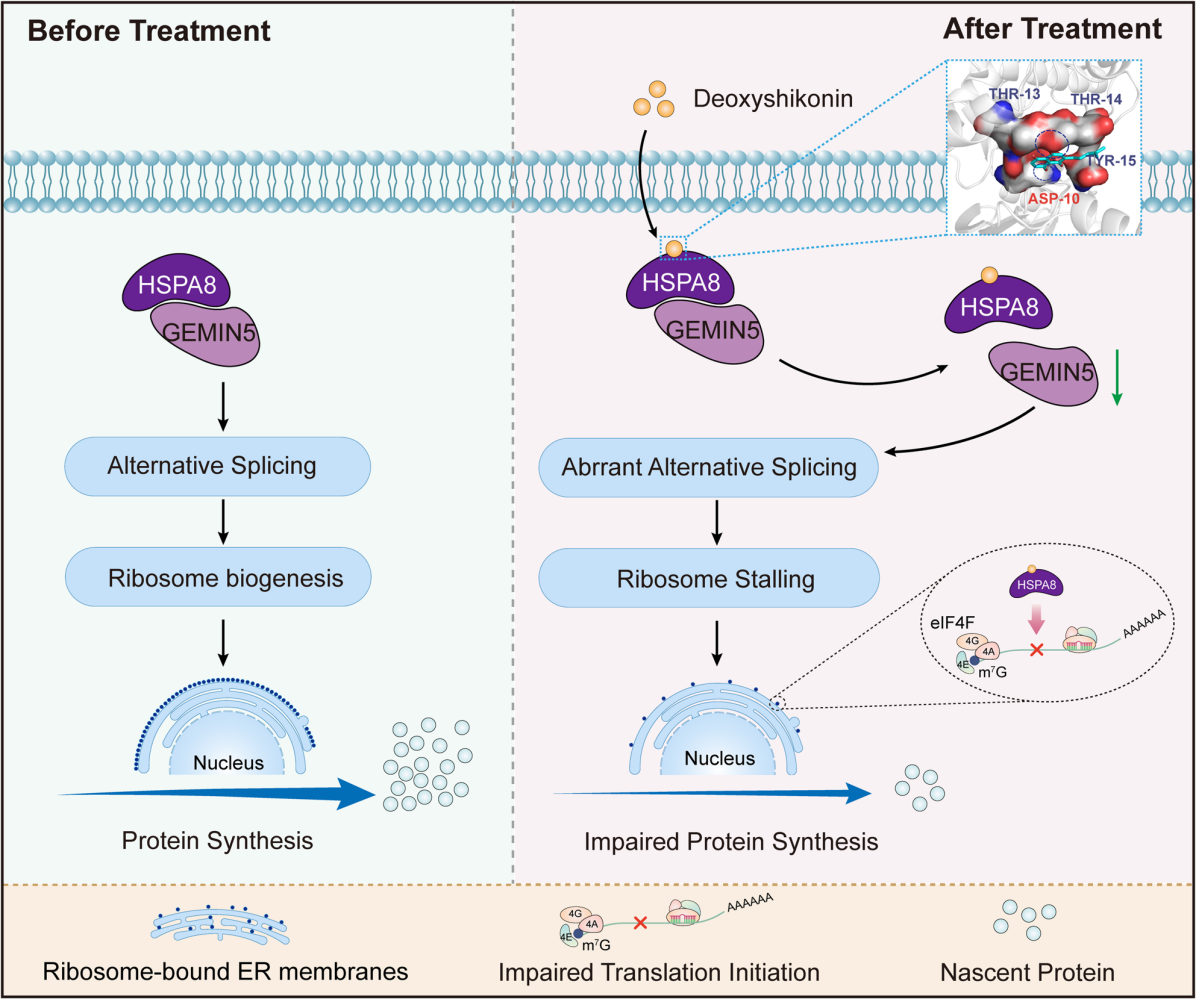

**Supplementary Information:**

The online version contains supplementary material available at 10.1186/s13046-026-03645-2.

## Background

Colorectal cancer (CRC) is among the most prevalent malignancies worldwide [1]. The reprogramming of proteostasis through aberrant protein synthesis in CRC cells is recognized as a core driver of malignant progression [2, 3]. Ribosome-targeting agents (e.g., CX-5461) show promise in hematological malignancies, although their efficacy in solid tumors remains limited due to insufficient precision in modulating tumor-specific translational pathways [4, 5]. Consequently, elucidating the molecular hubs governing protein synthesis dependency in CRC and developing selective intervention strategies are the critical unmet challenges in this field.

Heat shock protein family A member 8 (HSPA8), a core member of the HSP70 family, regulates protein synthesis initiation efficiency by stabilizing the structural conformation of eukaryotic translation initiation factor 4 gamma (eIF4G) beyond its functional capacity as a canonical chaperone [6, 7]. Gem nuclear organelle-associated protein 5 (GEMIN5), a component of the survival motor neuron (SMN) complex, participates in translation elongation complex assembly by directly binding with the ribosomal proteins, in addition to mediating pre-mRNA splicing [8]. This functional crosstalk suggests that HSPA8 and GEMIN5 might be involved in the cooperative remodeling of tumor proteostasis through the synergistic regulation of the splicing-translation coupling network. The mechanism underlying the interaction between HSPA8 and GEMIN5 in tumors and the regulatory impact of these two on ribosomal dynamics remains unexplored to date.

Alternative splicing (AS) is the core driver of proteomic diversity in tumors, and its dysregulation promotes cancer progression through the generation of oncogenic isoforms [9]. AS of the ribosomal protein (RP)-encoding genes has been reported as a novel regulatory mechanism in tumorigenesis [10]. However, current research in this field has focused primarily on copy number variations or expression dysregulation of RP genes, while the dynamic regulation of ribosomal function based on splicing patterns remains largely unexplored. Since HSPA8 and GEMIN5 are considered key molecules in splicing regulation (through HSPA8-splicing factor interactions) and translation quality control (through GEMIN5-ribosome interactions), studies need to assess whether HSPA8 and GEMIN5 cooperatively remodel the ribosomal dynamics by synergistically regulating the generation of RP splicing isoforms.

Based on literature research combined with screening of a small-scale antitumor natural compound library, we found that the natural compound Deoxyshikonin (DSHK) exhibits promising anti-CRC potential. However, in-depth investigations into the anti-CRC effects of DSHK are lacking. This study systematically elucidated the core mechanism of DSHK against CRC through a stepwise research strategy. First, the significant antitumor activity of DSHK in preclinical CRC models was demonstrated. HSPA8 was identified as the direct target of the anti-CRC action of DSHK. GEMIN5 was revealed as a novel functional interactor of HSPA8 in CRC. Moreover, it was revealed that DSHK treatment disrupts the functionality of the HSPA8-GEMIN5 complex, thereby triggering the aberrant splicing of ribosomal protein-encoding genes and impeding functional ribosome biogenesis. Further, DSHK was demonstrated to directly destabilize the HSPA8-eIF4F complex, inducing translational dysfunction. Collectively, these effects converge to disrupt proteostasis. This work is the first to identify the HSPA8-GEMIN5 complex as a molecular hub mediating “splicing-translation coupling” in CRC, and accordingly, a novel natural product anticancer paradigm of “targeting AS-ribosomal dysfunction” is proposed, providing fresh perspectives for developing CRC therapeutics.

## Materials and methods

### Reagents

Deoxyshikonin (DSHK; Catalog no. HY-N2187, purity ≥ 99%) was purchased from MedChemExpress (Monmouth Junction, NJ, USA), and a 10 mM stock solution was prepared by dissolving the compound in dimethyl sulfoxide (DMSO). The solution was stored at -20 °C under light-protected conditions. The remaining chemical reagents were purchased from Sigma-Aldrich (St. Louis, MO, USA), and the experimental consumables were sourced from Corning Incorporated (Corning, NY, USA).

### DSHK target fishing assay

Carboxyl magnetic beads (Candidate, Shanghai, China) were covalently conjugated to the phenolic hydroxyl groups of DSHK through DCC/DMAP-catalyzed esterification: 10 mg of beads were allowed to react with 20–40 mmol of DSHK and the catalysts in anhydrous dichloromethane for 3 h. The reaction mixture was then sequentially washed with acid/base solutions, followed by drying and purification through silica gel column chromatography (CH_2_Cl_2_ elution) to obtain the beads-DSHK conjugates. After blocking with casein/PBST, these conjugates were stored at 4 °C. The target protein capture system included three experimental sets: an experimental group (2 mg of conjugates + 800 µL of cell lysate), a bare bead control, and a drug competition control (preincubation with free DSHK). After overnight incubation at 4 °C with agitation, the beads were separated using a magnetic rack, washed with PBS, and resuspended before boiling denaturation. SDS-PAGE (30 µL loading mixture) was performed, and specific bands of the experimental group were identified, excised, enzymatically digested, and then desalted using C18 columns. The samples were then analyzed on a Thermo HF-X mass spectrometer (analysis column: 75 μm × 20 cm C18-AQ 1.9 μm). The high-confidence target proteins, screened using the MaxQuant and Perseus software. Quality control confirmed the conjugation success through zeta potential measurements: bare beads (-37.1 mV) vs. DSHK-conjugated beads (-25.9 mV).

### Specific Pupylation as IDEntity Reporter (SPIDER) proximity labeling technology​

The SPIDER proximity labeling technique was applied to screen the HSPA8-interacting proteins. Biotinylated HSPA8 protein (0.1 µM) was incubated overnight with 3 mg of cell lysate in a reaction buffer (final volume of 6 mL) at 4 °C. After the addition of 0.1 µM SA-Pup4-labeling enzyme (abcode, Shanghai, China), the reaction was allowed to proceed at room temperature for 20 min, followed by supplementation with 5 mM ATP and 0.25 µM PafA. The mixture was rotationally incubated at 37 °C for 30 min to activate biotinylation. The reaction system was then denatured using urea (8 M final concentration), after which 0.1% Tween-20 and 100 µL of streptavidin agarose beads were added for a 2 h incubation at room temperature to capture the biotinylated protein complexes. Centrifugation was performed to remove the supernatant, and the precipitate was washed three times with 14 mL of the washing buffer (through centrifugation at 1,500 rpm with 5 min of room temperature rotation per cycle). The enriched interaction protein complexes were digested with trypsin and analyzed using a Thermo HF-X mass spectrometer (analysis column: 75 μm × 20 cm C18-AQ 1.9 μm). Data processing using the MaxQuant software (v1.6.0.1) identified high-confidence interacting proteins meeting the criteria of ≥ 2 unique peptides and a fold change ≥ 1.2 in replicate experiments, excluding the common contaminants such as ribosomal proteins and keratins.

### Surface Plasmon Resonance (SPR) assay

SPR analysis was performed using a Biacore T200 system (GE Healthcare). The CM5 sensor chip was activated using EDC/NHS, followed by immobilization of the HSPA8 protein (diluted to 50 µg/mL in sodium acetate buffer, pH 4.0) onto flow cell 4 at a flow rate of 10 µL/min (coupling level: 9112.9 response units, RUs). The chip surface was subsequently blocked using ethanolamine hydrochloride, with flow cell 3 serving as a blank reference. DSHK was then serially diluted in the PBS-P^+^ buffer (pH 7.4) containing 5% DMSO, and solvent calibration curves were generated for correction. Gradient concentrations of DSHK were injected over the chip surface at a 30 µL/min flow rate with a 150-s association time. After each injection, the chip was regenerated with 10 mM glycine-HCl (pH 2.0) for 5 min. Real-time sensorgrams were reference-subtracted, and the binding kinetics (association rate constant (Ka), dissociation rate constant (Kd), and equilibrium dissociation constant (K_D_)) were determined using global fitting to a 1:1 Langmuir binding model in BIAcore T200 evaluation software v2.0.

### Native polyacrylamide gel electrophoresis (Native PAGE)

The samples were mixed with 4X native loading buffer (62.5 mM Tris-HCl, pH 6.8, 40% glycerol, 0.01% bromophenol blue) in a 3:1 ratio, strictly avoiding heating, reducing agents, or denaturants. A 6%-12% separating gel (acrylamide: bis-acrylamide = 29:1, 0.375 M Tris-HCl, pH 8.8) and a 4% stacking gel (pH 6.8) were prepared. After the electrophoresis tank was precooled to 4 °C, prechilled running buffer (25 mM Tris, 192 mM glycine, pH 8.3) was added. The samples (20–30 µL per lane) were loaded, and electrophoresis was conducted at 4 °C and 80 V constant voltage applied across the stacking gel (∼30 min until the dye migrated to the separating gel interface), followed by 120 V applied across the separating gel (90 min or until the dye reached the gel bottom). After the electrophoresis, the proteins were transferred to PVDF membranes through a semidry transfer (200 mA constant current, 90 min, 4 °C). The membranes were blocked with 5% skim milk at room temperature for 1 h, and then sequentially incubated with primary antibodies (overnight at 4 °C) and HRP-conjugated secondary antibodies (1 h at room temperature). Afterward, the membranes were thoroughly washed with TBST and developed with an ECL substrate in the dark. The exposure times were manually optimized based on the signal intensity.

### Puromycin-mediated nascent chain labeling assay

The cells were placed in a culture medium containing 10 µg/mL puromycin for 30 min. After the medium was discarded, the cells were gently washed twice with prechilled PBS to remove the residual drug. Precooled RIPA lysis buffer supplemented with 1× protease inhibitor cocktail was added for 30 min of lysis on ice. The lysates were subsequently centrifuged at 12,000 ×g for 15 min at 4 °C to remove cellular debris. The collected supernatants were mixed with 4× SDS loading buffer and denatured by boiling at 100 °C for 5 min. The prepared samples were then used for the western blot analysis.

### Sucrose density gradient centrifugation assay

A linear 10%-50% (w/v) sucrose density gradient solution containing 20 mM HEPES-KOH (pH 7.4), 100 mM KCl, 5 mM MgCl_2_, and 1 mM DTT was prepared. Continuous gradients were prepared in ultracentrifuge tubes (Beckman, 14 × 89 mm) using a gradient former followed by equilibration at 4 °C for 2 h to eliminate interfacial disturbances. The cell lysates were subjected to differential centrifugation at 12,000 ×g (4 °C, 20 min) to remove the mitochondria and cell debris. Subsequently, 800 µL of the clarified supernatant was gently layered onto the gradient surface without vortexing, and centrifugation was performed at 40,000 rpm for 2 h at 4 °C using a Beckman Optima XE-90 ultracentrifuge with an SW41Ti rotor. Afterward, ten 1 mL fractions were collected through bottom puncture (18G needle), and their UV absorbance at 254 nm was monitored in real time using a BioLogic LP chromatography system (bandwidth: 2 nm, sampling rate: 1 Hz). A_254_ peaks were recorded to identify the characteristic sedimentation positions (40 S, 60 S subunits, and 80 S monosomes). The collected fractions were concentrated using Amicon Ultra 100 kDa centrifugal filters to remove sucrose for subsequent subunit protein quantification.

### Luciferase thermotolerance refolding assay

The cells transfected with the pRSV-luciferase plasmid for 24 h were seeded in the wells of 12-well plates (5 × 10^4^ cells/well) and pretreated with 100 µg/mL cycloheximide for 30 min to inhibit nascent protein synthesis. Heat shock induction was performed by sealing the plates in a precision-controlled 45 °C water bath for 30 min, followed by rapid transfer to a 37 °C incubator for different recovery periods (0, 1, 2 h). At each time point, the cells were washed with ice-cold PBS and lysed in 100 µL of buffer (25 mM Tris-HCl pH 7.8, 2 mM DTT, 1% Triton X-100) on ice for 15 min. The lysates were then centrifuged at 12,000 ×g (4°C, 10 min), and 20 µL of the supernatant was mixed with an equal volume of luciferase substrate (Luciferase Assay System, Promega). Relative luminescence units (RLUs) were immediately measured using a microplate reader. The reactivation rates (%) were calculated relative to those of the 0 h recovery group, and the data were normalized to the total protein concentration determined using the BCA assay. Untransfected cells and 42 °C heat shock controls were included for specificity validation.

### Statistical analysis

All experiments were repeated independently a minimum of three times. The data were then presented as mean ± standard deviation (SD). Differences between the experimental and control groups were determined using analysis of variance (ANOVA) and Student’s *t* test, with *P* < 0.05 considered statistically significant. All statistical analyses were performed using SPSS 17.0 software (IBM Corp., USA).

## Results

### DSHK demonstrates significant antitumor activity in preclinical CRC models

**Fig. 1:**
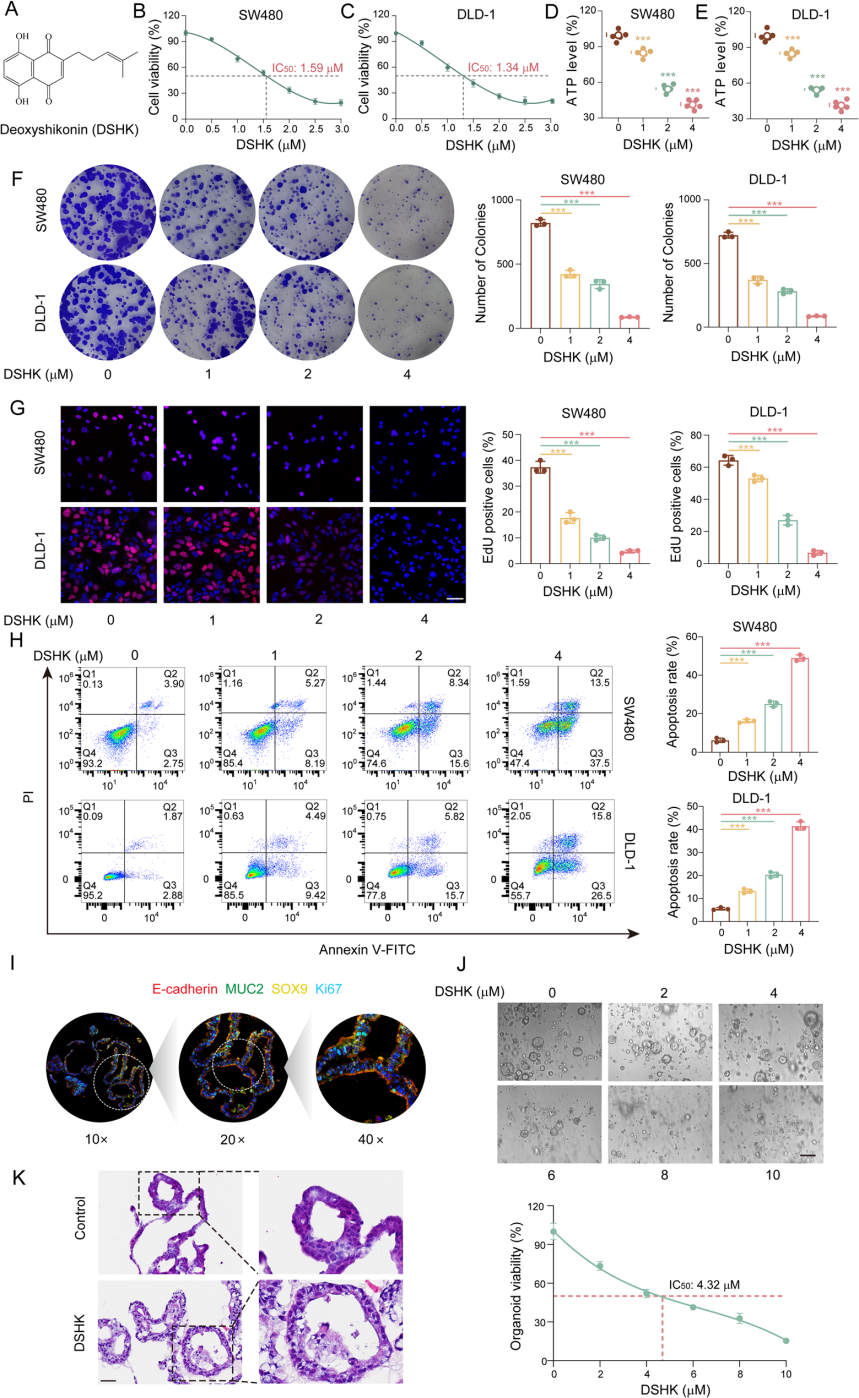
The in vitro anti-CRC activity of DSHK. **A** Chemical structure of DSHK; (**B**-**C**) Dose-dependent inhibition of cell viability in human CRC SW480 and DLD-1 cells, measured using a CCK-8 assay; (**D**-**E**) Intracellular ATP content quantification using the bioluminescence assay after 24 h of DSHK treatment; (**F**) Colony formation assay for the analysis of the DSHK-mediated suppression of cell proliferation in SW480 and DLD-1 cells; (**G**) EdU fluorescence staining to detect the DNA replication activity after 24 h of DSHK exposure (scale bar = 50 μm); (**H**) Annexin V-FITC/PI dual-staining flow cytometry to quantify the DSHK-induced apoptosis after 24 h of treatment; (**I**) Multiplex immunofluorescence staining of E-cadherin/MUC2/Ki67/SOX9 in the PDOs (scale bar = 100 μm); (**J**) ATP activity detection and microscopic morphology of the PDOs after gradient DSHK treatment for 7 days (scale bar = 100 μm); (**K**) H&E-stained histopathological features of the PDOs after 7 days of treatment with DSHK (8 µM) (scale bar = 25 μm). The data presented are the mean ± SD of at least three independent experiments (^***^P < 0.001)

This study systematically evaluated the cytotoxic effects of DSHK on CRC utilizing a dual-dimensional “toxicity-efficacy” assessment framework. The human normal colon epithelial cell line NCM460 served as a reference for toxicity, whereas seven human CRC cell lines (HCT-116, RKO, SW480, DLD-1, Caco-2, SW620, and HT-29) were used as models for efficacy. The experimental findings indicated that the 48 h IC_50_ value of DSHK for NCM460 cells exceeded 40 µM. In contrast, the IC_50_ values for seven CRC cell lines tested was less than 5 µM (Supplementary Fig. S1). These results indicated that DSHK demonstrated good tumor-selective cytotoxicity against CRC. Moreover, dose-response experiments in seven CRC cell lines revealed that SW480 and DLD-1 cells were the most sensitive to DSHK treatment, with corresponding IC_50_ values of 1.59 µM and 1.34 µM, respectively (Fig. 1B-C). Consequently, human CRC SW480 and DLD-1 cells were selected for further investigation of the anti-CRC mechanisms of DSHK.

Next, ATP activity assays were performed, which revealed that DSHK downregulated the ATP levels in both SW480 and DLD-1 cells in a concentration-dependent manner, indicating marked reduction in the cellular energy metabolism (Fig. 1D-E). Clonogenic and EdU staining assays demonstrated that DSHK could significantly suppress the proliferative capacities of SW480 and DLD-1 cells (Fig. 1F-G). Flow cytometric analysis revealed that DSHK potently induced apoptosis in the SW480 and DLD-1 cells (Fig. 1H). Furthermore, to comprehensively evaluate the anti-CRC efficacy of DSHK, a patient-derived CRC organoid (PDO) model was established (Supplementary Fig. S2A-B), and these organoids presented characteristic CRC features, as validated through the biomarker expression profiles and morphological confirmation (Fig. 1I, Supplementary Fig. S2C-D). DSHK treatment of the PDOs yielded an IC_50_ value of 4.32 µM (Fig. 1J). Moreover, the H&E staining results revealed distinct apoptotic features, including nuclear pyknosis and fragmentation, in the DSHK-treated organoids (Fig. 1K).

**Fig. 2:**
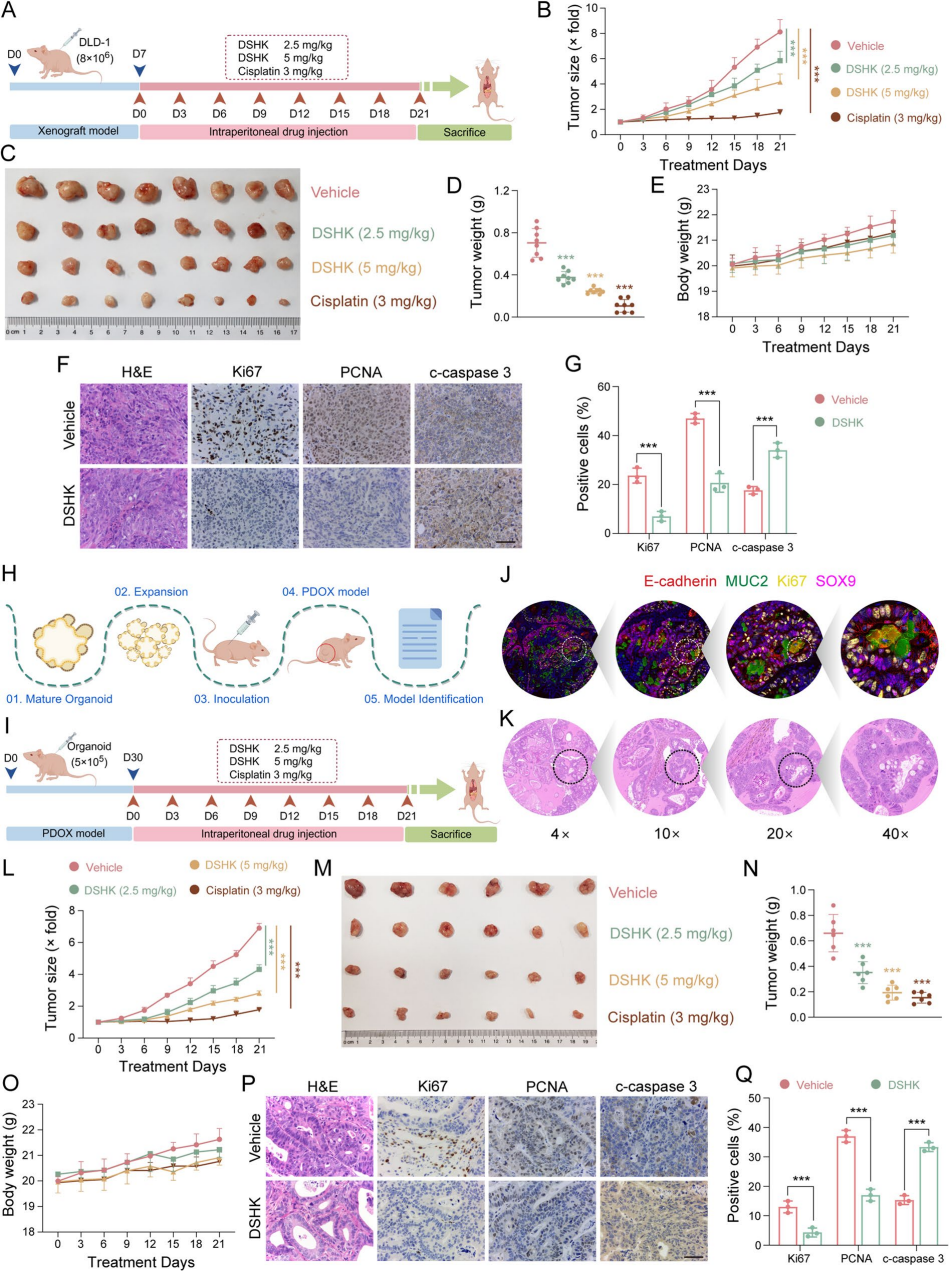
The in vivo anti-CRC efficacy of DSHK. **A** CDX model establishment: Nude mice subcutaneously inoculated with DLD-1 cells received the intraperitoneal injections of positive controls (cisplatin, 3 mg/kg) or DSHK (2.5/5 mg/kg) every 2 days for 21 days post-inoculation, *n* = 8; (**B**-**E**) Dynamic tumor volume monitoring, tumor weight measurement, and body weight tracking for the CDX model; (**F**) H&E staining and IHC of the CDX tumors to assess the proliferation markers (Ki67/PCNA) and apoptosis markers (cleaved-caspase 3); (**G**) Quantitative analysis of the IHC signals in the CDX tumors; (**H**-**I**) PDOX model construction, performed following inoculation/dosing protocols identical to those adopted for the CDX model, *n* = 6; (**J**) Multiplex immunofluorescence localization of E-cadherin, MUC2, Ki67, and SOX4 in the PDOX tissues; (**K**) H&E staining results confirmed the histomorphological characteristics of PDOX tumors; (**L**-**O**) Tumor volume monitoring, tumor weight recording, and body weight changes in the PDOX model; (**P**) H&E and IHC staining of the PDOX tumors (targets identical to those in panel F); (**Q**) Quantitative IHC analysis of the PDOX tumors. Scale bars = 25 μm for all pathological staining data. The data presented are the mean ± SD of at least three independent experiments (^***^P < 0.001)

In order to further evaluate the in vivo anti-CRC activity of DSHK, cell line-derived xenograft (CDX) models (Fig. 2A) and patient-derived organoid-based xenograft (PDOX) models (Fig. 2H-I) were established. These PDOX models exhibited characteristic CRC features, as validated through the biomarker expression profiles and morphological confirmation (Fig. 2J-K). In both the CRC animal models, DSHK administration significantly suppressed tumor growth, and the high-dose groups achieved tumor inhibition rates of 64.36% in CDX and 70.32% in PDOX models (Fig. 2B-D and L-N). Histopathological analysis of the major organs and body weight monitoring revealed no significant toxicity in the DSHK-treated groups (Fig. 2E and O; Supplementary Fig. S3). Furthermore, pathological examination revealed nuclear atypia and architectural disorganization in DSHK-treated tumors, which was accompanied by the downregulation of proliferation markers (Ki67, PCNA) and the upregulation of the apoptosis marker cleaved-caspase 3 (Fig. 2F-G and P-Q), confirming the in vivo anti-CRC efficacy of DSHK.

### HSPA8 as the direct target of DSHK against CRC​

**Fig. 3:**
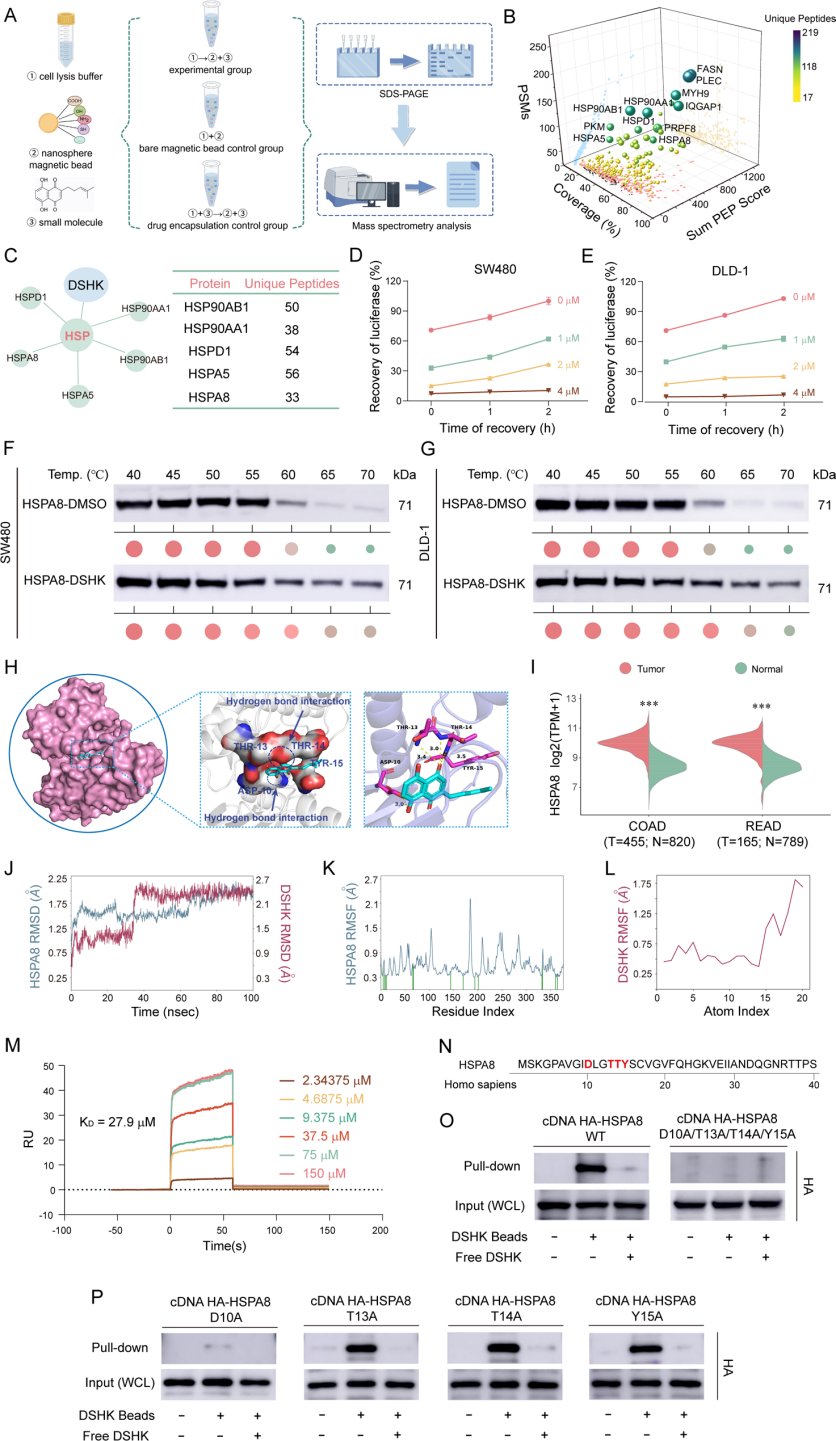
HSPA8 is a direct target of DSHK against CRC. **A** Experimental workflow of the chemical proteomics for the identification of DSHK targets against CRC; (**B**) Three-dimensional projection analysis of target enrichment using five-dimensional parameters (sum PEP score, coverage, peptides, PSMs, and unique peptides): Sphere color map unique peptide abundance and sphere volume map peptide count; (**C**) Functional enrichment analysis of the HSP family in the chemical proteomics dataset: Sphere volume map unique peptide abundance; (**D**-**E**) Firefly luciferase thermotolerance refolding assay: Luciferase-transfected cells (24 h) subjected to heat recovery treatment for the quantification of protein refolding activity; (**F**-**G**) CETSA validation of the thermal stability of the drug-target complex; (**H**) Molecular docking simulation of the DSHK-HSPA8 binding mode; (**I**) Transcriptomic data mining of HSPA8 expression in the TCGA CRC cohort; (**J**-**L**) Molecular dynamics simulation (Desmond 4.2, 100 ns) to quantify complex conformational stability: Root mean square deviation (RMSD) and atomic fluctuation (RMSF) calculations; (**M**) SPR-based determination of the DSHK-HSPA8 binding dissociation constant (K_D_); (**N**-**P**) Mutational analysis of DSHK-HSPA8 interaction specificity in the HEK293T cells transiently transfected with wild-type or mutant HSPA8 plasmids (48 h), using the GST pull-down assay. Data presented are the mean ± SD of at least three independent experiments (^***^P < 0.001)

In order to elucidate the direct target of DSHK in CRC, an integrated screening strategy combining microsphere capture, chemical modification, and mass spectrometry was implemented (Fig. 3A). Efficient target capture was achieved through the esterification reaction between the hydroxyl groups of DSHK and the carboxyl-modified microspheres (Supplementary Fig. S4). The candidate targets were comprehensively ranked based on their binding abundance, specificity scores, and enrichment fold changes (Fig. 3B). Preliminary validation of the top 12 high-confidence targets using CETSA and molecular docking and functional enrichment analysis revealed members of the heat shock protein family as potential targets for the anti-CRC activity of DSHK (Supplementary Fig. S5A, Supplementary Fig. S5B, and Fig. 3C).

Further investigations were performed using heat shock refolding functional assays, which revealed that DSHK impaired the functional recovery capacity of heat shock proteins (HSPs) in a concentration- and time-dependent manner (Fig. 3D-E). This finding confirmed the direct interference of DSHK with the heat shock pathway. Therefore, we further focused on the members of the HSP family (HSP90, HSPA8, HSPD1, and HSPA5). The CETSA results showed that DSHK treatment significantly enhanced the thermal stability of HSPA8, while it had no significant effect on the thermal stability of HSP90, HSPD1, or HSPA5 (Fig. 3F-G, Supplementary Fig. S5A). Moreover, molecular docking results revealed that DSHK exhibited the strongest binding affinity to HSPA8 compared with other HSP family members (HSPA8 binding energy ΔG = -7.9 kcal/mol, HSP90 binding energy ΔG = -6.6 kcal/mol, HSPD1 binding energy ΔG = -6.9 kcal/mol, HSPA5 binding energy ΔG = -7.1 kcal/mol), and it formed a hydrogen bond network with HSPA8 through the residues ASP10, THR13, THR14, and TYR15 (Fig. 3H, Supplementary Fig. S5B). Therefore, HSPA8 was ultimately selected as the core candidate target of DSHK. Bioinformatics analysis was conducted using the data from the TCGA database, which revealed significantly elevated HSPA8 mRNA expression in human CRC tissues compared to the adjacent normal tissues (Fig. 3I), and positive correlations were noted between HSPA8 expression and the expressions of tumor proliferation/apoptosis markers (Supplementary Fig. S6A-C). Kaplan-Meier survival analysis revealed significantly shorter overall survival in CRC patients with high HSPA8 expression (Supplementary Fig. S6D). Subsequent 100 ns molecular dynamics simulations revealed that the DSHK-HSPA8 complex maintained high conformational stability, with post-equilibrium root mean square deviation (RMSD) fluctuations < 0.4 nm (Fig. 3J) and low root mean square fluctuation (RMSF) values (Fig. 3K-L). SPR assays revealed a binding constant K_D_ of 27.9 µM for the DSHK-HSPA8 interaction (Fig. 3M), indicating specific binding. Functional analysis confirmed the critical role of these binding residues in maintaining the functions of HSPA8 protein (Supplementary Fig. S7). Mutational analysis was performed next, and it was revealed that in pull-down assays, simultaneous alanine substitutions at ASP10, THR13, THR14, and TYR15 (D10A/T13A/T14A/Y15A) abolished the DSHK-HSPA8 binding (Supplementary Fig. S7A, Fig. 3N-O). Notably, a single-point mutation at ASP10 (D10A) completely disrupted this interaction (Fig. 3P), and this finding established ASP10 as the core binding site.

### Anti-CRC activity of DSHK depends on HSPA8

**Fig. 4:**
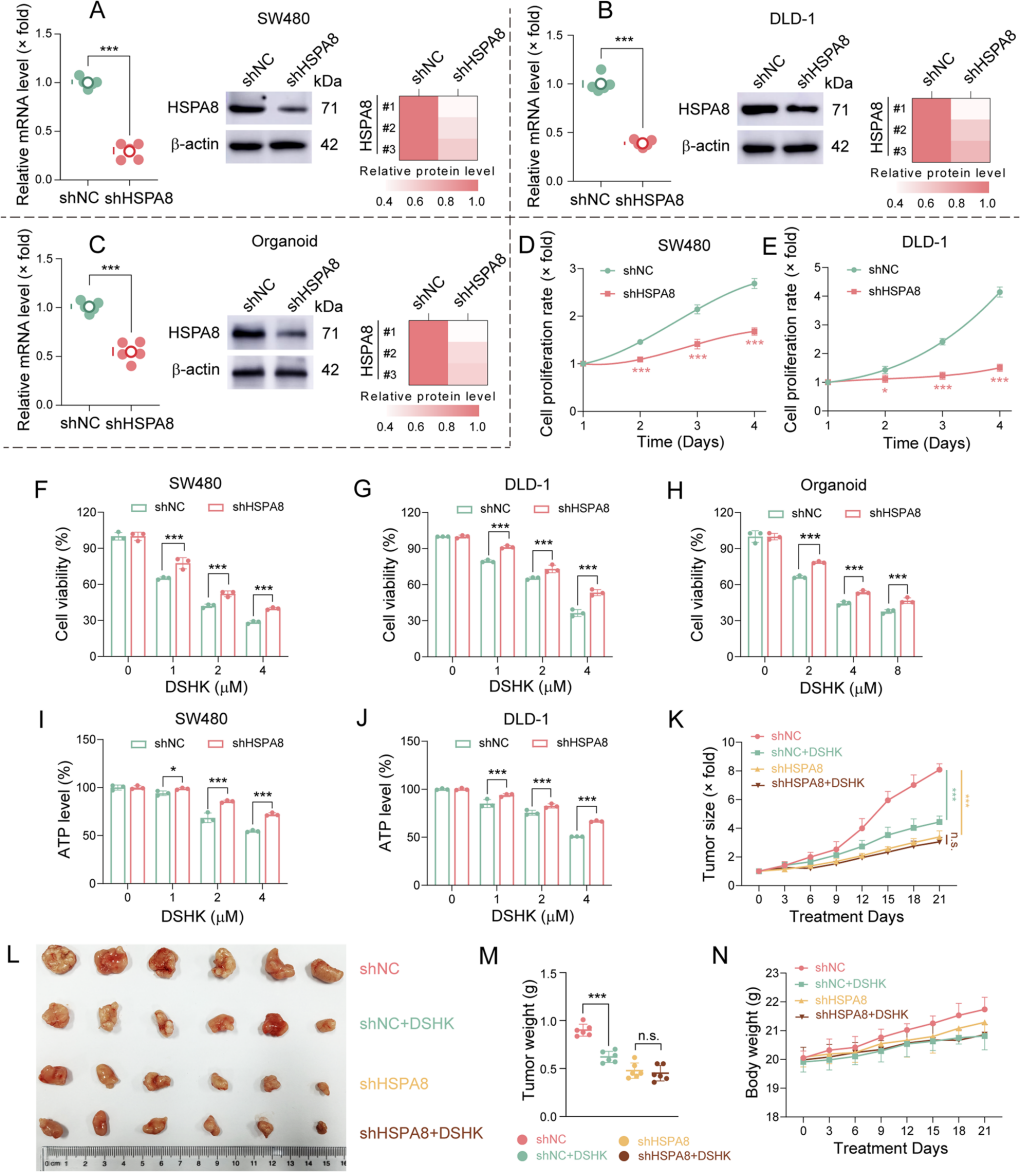
Dependence of DSHK anti-CRC activity on HSPA8. **A**-**C** Analysis of the changes in the HSPA8 mRNA and protein expression in SW480/DLD-1 cells and the organoids following lentiviral shRNA-mediated knockdown, as assessed using qRT-PCR and western blotting; (**D**-**E**) Proliferation kinetics of the shHSPA8-transfected SW480/DLD-1 cells determined using the CCK-8 assay; (**F**-**H**) Quantitative CCK-8 analysis of the survival rates of SW480/DLD-1 cells and organoids after DSHK treatment (24 h) or shHSPA8 knockdown; (**I**-**J**) Metabolic activity detection in SW480/DLD-1 cells under DSHK treatment (24 h) or shHSPA8 silencing, using the ATP bioluminescence assay; (**K**-**N**) Tumor growth dynamics in BALB/c nude mice subcutaneously injected with stable DLD-1-shHSPA8 cells: DSHK (5 mg/kg) was administered intraperitoneally every 2 days starting from Day 7 post-inoculation for 21 days, with shNC + DSHK. The data presented are the mean ± SD of at least three independent experiments (^*^P < 0.05 and ^***^P < 0.001)

In order to investigate whether the anti-CRC activity of DSHK depends on HSPA8, HSPA8 knockdown was achieved in SW480 and DLD-1 cells using siRNAs. HSPA8 knockdown significantly attenuated the sensitivity of the cells to DSHK treatment (Supplementary Fig. S8). Furthermore, shHSPA8 lentiviral systems were successfully constructed, and using these, stable knockdown models were established in SW480/DLD-1 cells and PDOs (Supplementary Fig. S9A-B, Fig. 4A-C). HSPA8 depletion markedly suppressed the proliferation of SW480/DLD-1 cells (Fig. 4D-E). Moreover, shRNA-mediated HSPA8 knockdown substantially decreased the inhibitory effects of DSHK on cell viability in both cell lines and organoids (Fig. 4F-H, Supplementary Fig. S9C). ATP assays and clonogenic experiments confirmed that HSPA8 deficiency could significantly impair DSHK-induced proliferation blockade in CRC cells (Fig. 4I-J, Supplementary Fig. S9D). Given the cooperative chaperone activity between HSPA8 and HSP90 [11, 12], the potential impact of DSHK on HSPA8 function was investigated indirectly by analyzing its regulation of the HSP90 client protein β-catenin. As shown in Supplementary Fig. S9F-I, DSHK promoted β-catenin degradation via the ubiquitin-proteasome pathway. In order to evaluate the role of HSPA8 on the in vivo anti-CRC effects of DSHK, xenograft tumors were retrieved from stable HSPA8-knockdown DLD-1 cells, followed by DSHK administration. Figure 4O-R showed that HSPA8 knockdown significantly inhibited tumorigenicity in vivo, with no significant difference in tumor volume or weight between the shHSPA8 and shHSPA8 + DSHK groups, indicating that HSPA8 ablation abrogated the antitumor efficacy of DSHK in vivo. Collectively, these results confirmed that the anti-CRC effects of DSHK depend on HSPA8.

### GEMIN5 is a novel functional interactor of HSPA8

**Fig. 5:**
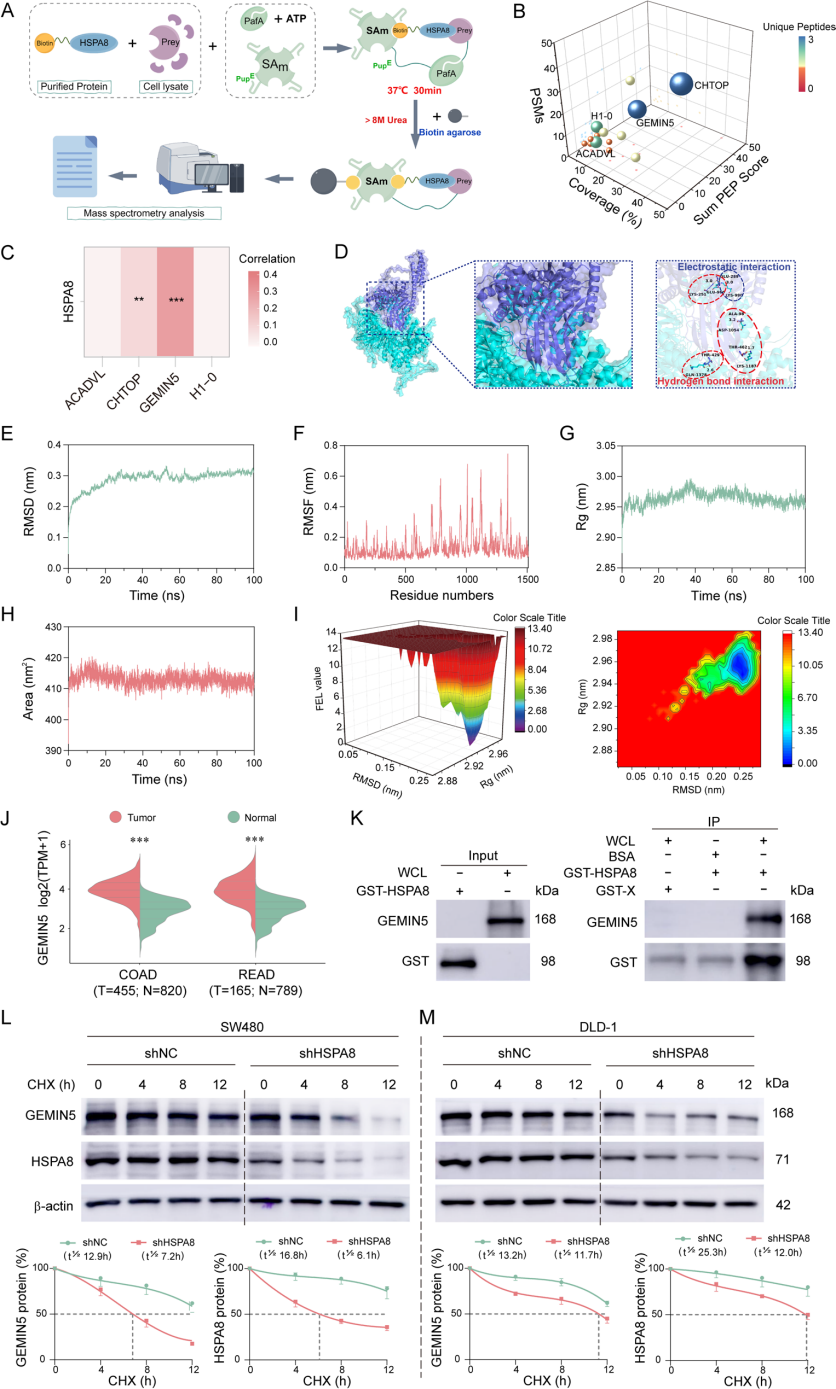
GEMIN5 as a novel functional interactor of HSPA8. **A** SPIDER-based workflow for the systematic screening of the HSPA8 interactome (biotin proximity-labeling covalent capture strategy); (**B**) Three-dimensional projection of target enrichment using five-dimensional parameters (sum PEP score, coverage, peptides, PSMs, and unique peptides): Sphere color maps of the relative abundance of the unique peptides and sphere volume maps of peptide count; (**C**) Genomic coexpression correlation between HSPA8 and the candidate interactors (4 proteins) mined from the TCGA database; (**D**) Molecular docking simulation-based prediction for the HSPA8-GEMIN5 binding mode; (**E**-**I**) Molecular dynamics simulation of the HSPA8-GEMIN5 complex (Gromacs 2020, 100 ns): Conformational stability (RMSD/RMSF), spatial dynamics (radius of gyration Rg), SASA, and free energy landscape; (**J**) Transcriptomic profiling of GEMIN5 expression in the TCGA CRC cohort; (**K**) GST pull-down validation of the HSPA8–GEMIN5 interaction in HEK293T lysates; (**L**-**M**) Cycloheximide (CHX, 10 µM) chase assay to quantify the impact of HSPA8 knockdown on the GEMIN5 protein half-life in SW480/DLD-1 cells. The data presented are the mean ± SD of at least three independent experiments (^**^P < 0.01, and ^***^P < 0.001)

Based on the molecular chaperone functional characteristics of HSPA8, this study employed the novel SPIDER proximity labeling technique and a covalent biotin labeling strategy to capture HSPA8-interacting proteins specifically. Through rigorous mass spectrometry analysis and crosslinking validation, we identified the chromatin targets of PRMT1 (CHTOP), GEMIN5, H1 histone family member 0 (H1-0), and acyl-CoA dehydrogenase, very long chain (ACADVL) as four high-confidence covalently bound proteins of HSPA8 (Fig. 5A-B, Supplementary Fig. S10A-B). Correlation analysis and molecular docking results were used to predict the optimal binding energy for the HSPA8-GEMIN5 interaction, which featured three critical hydrogen bonds and strong electrostatic complementarity at the interface (Fig. 5C-D, Supplementary Fig. S10C-D). TCGA database analysis revealed that GEMIN5 overexpression in CRC was positively correlated with tumor proliferation and could predict poor prognosis (Fig. 5J, Supplementary Fig. S11). Molecular dynamics simulations demonstrated the high structural stability of the HSPA8-GEMIN5 complex: RMSD fluctuations < 0.4 nm (Fig. 5E), low global RMSF values (Fig. 5F), and a narrow range of fluctuations in the radius of gyration (Rg, 2.92–2.98 nm) and solvent accessible surface area (SASA, 410–420 nm^2^), confirming tight binding (Fig. 5G-H). Free energy landscape analysis revealed a single smooth energy basin with a global minimum at Rg = 2.94–2.98 nm and RMSD = 0.22–0.27 nm, collectively indicating a compact and stable nanoscale interaction architecture (Fig. 5I). The GST pull-down assays confirmed that recombinant HSPA8 specifically enriched GEMIN5 (Fig. 5K). HSPA8 knockdown significantly shortened the GEMIN5 protein half-life in SW480/DLD-1 cells (Fig. 5L-M). Collectively, these results established GEMIN5 as a novel functional interactor of HSPA8.

### DSHK disrupts the HSPA8-GEMIN5 interaction​

**Fig. 6:**
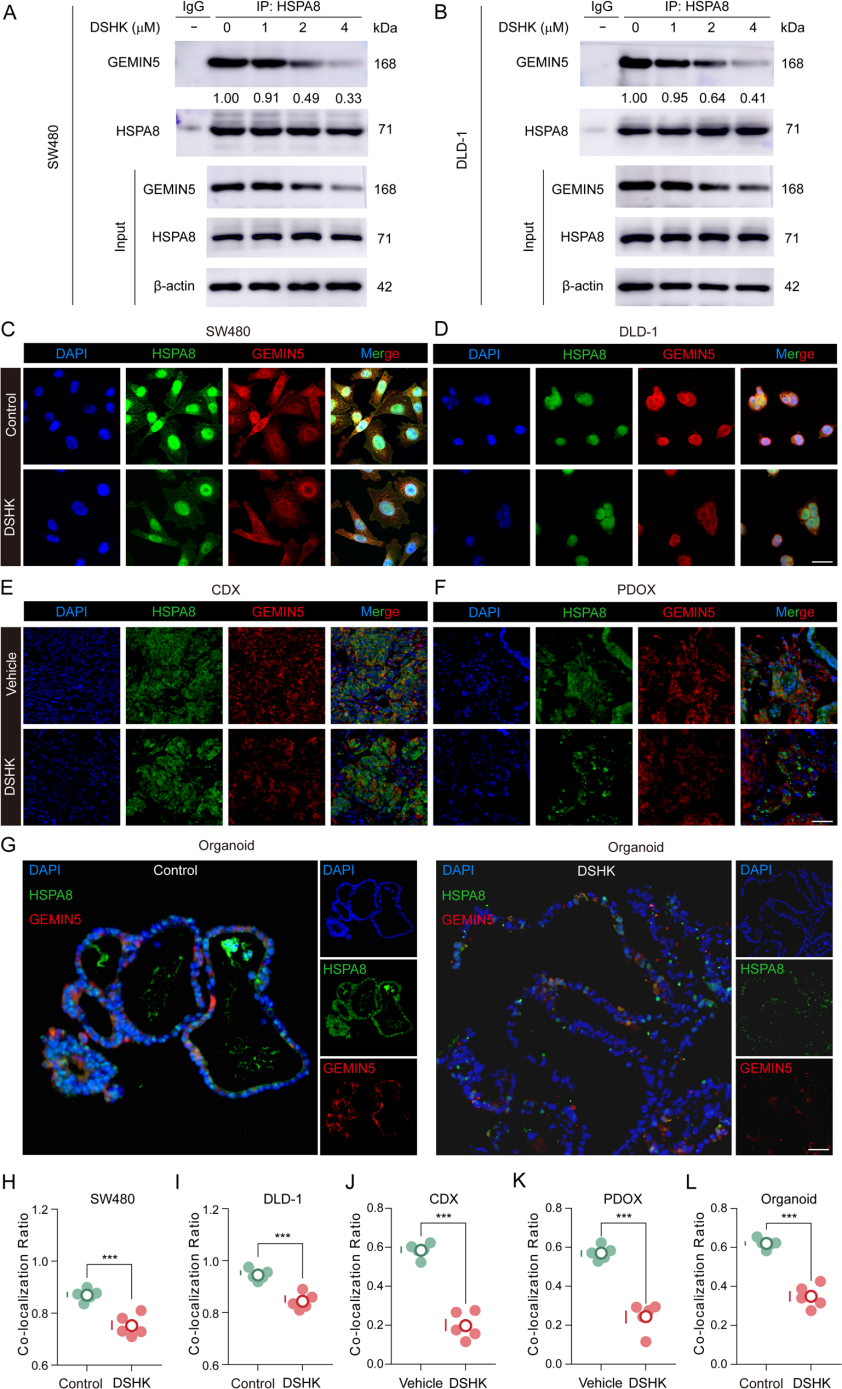
DSHK disrupts the HSPA8-GEMIN5 interaction. **A**-**B** Endogenous Co-IP analysis of the HSPA8-GEMIN5 interaction intensity in SW480 and DLD-1 cells after treatment with gradient concentrations of DSHK (24 h); (**C**-**L**) Subcellular colocalization analysis across the triple model systems under DSHK intervention: Cellular model: human CRC SW480 and DLD-1 cells (4 µM DSHK, 24 h; scale bar = 25 μm). Xenograft model: CDX/PDOX tumor-bearing mice (5 mg/kg DSHK; scale bar = 50 μm). Organoid model: PDOs (8 µM DSHK, 7 days; scale bar = 150 μm). Images acquired using laser confocal microscopy; Pearson’s colocalization coefficients were calculated using Image-Pro Plus software. The data presented are the mean ± SD of at least three independent experiments (^***^P < 0.001)

Whether DSHK treatment destabilizes the HSPA8-GEMIN5 complex was investigated next in this study. Co-immunoprecipitation (Co-IP) assays confirmed that HSPA8 and GEMIN5 formed a stable complex, and that DSHK significantly attenuated their binding affinity (Fig. 6A-B). Furthermore, subcellular colocalization analysis revealed high spatial overlap between HSPA8 and GEMIN5 in the perinuclear regions across the three model systems: SW480/DLD-1 cells, organoid models, and murine tumor tissues. DSHK treatment substantially reduced the degree of HSPA8-GEMIN5 colocalization, as evidenced by the significantly decreased Pearson’s correlation coefficients (Fig. 6C-L). Collectively, these results demonstrated the DSHK-mediated disruption of the HSPA8-GEMIN5 protein-protein interaction.

### Clinical relevance of HSPA8 and GEMIN5 expressions and interaction in CRC​

**Fig. 7:**
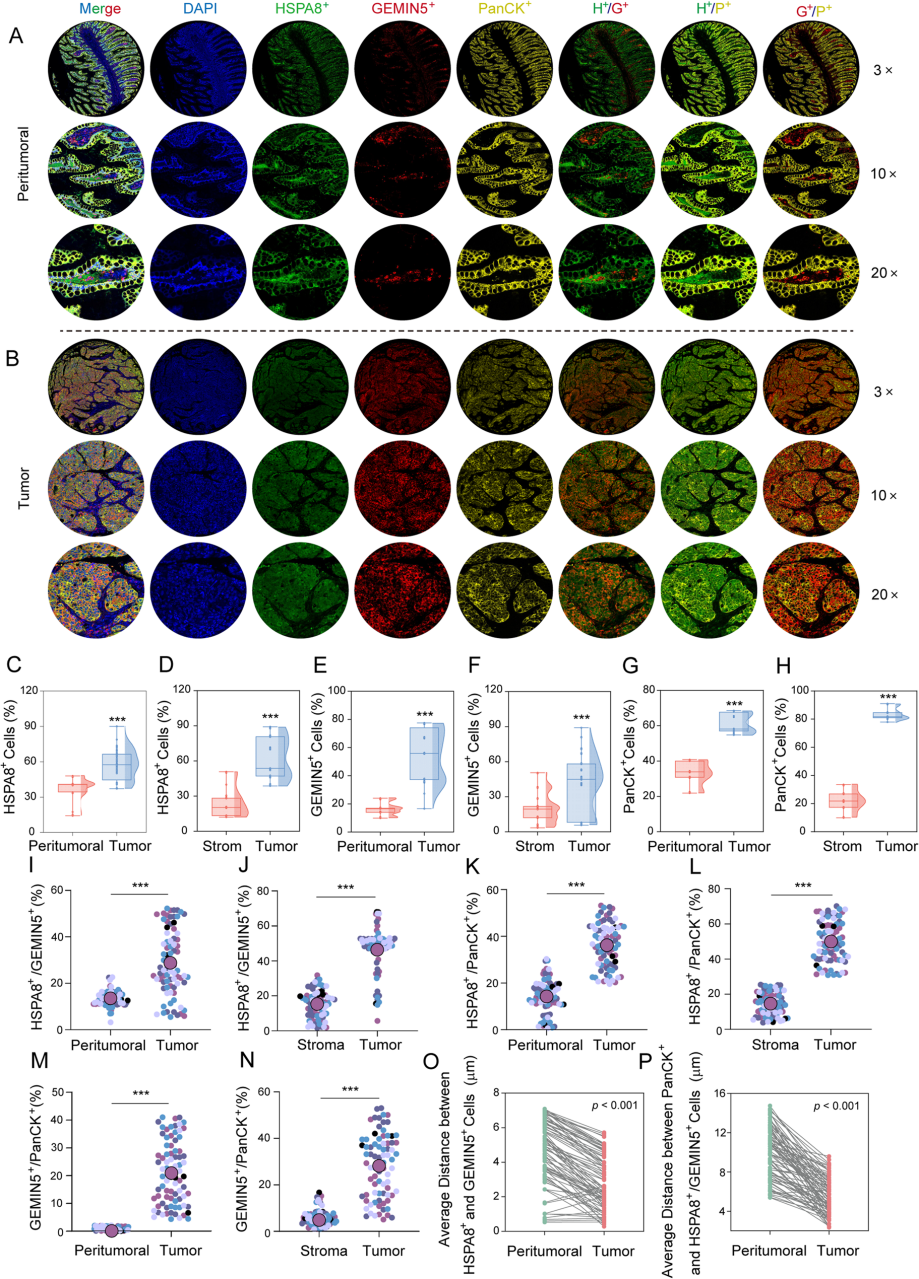
Expression, interaction, and colocalization analysis of HSPA8 and GEMIN5 in CRC clinical specimens. Using 80 paired CRC/adjacent normal TMAs, triple immunofluorescence staining was performed to simultaneously label the following proteins: HSPA8 (Alexa Fluor 488, green), GEMIN5 (Alexa Fluor 555, red), and PanCK (Alexa Fluor 647, yellow). **A**-**B** Representative multiscale confocal microscopy images (3 ×, 10 ×, and 20 × magnification fields) of the samples; (**C**-**H**) Fluorescence intensity distributions of HSPA8, GEMIN5, and PanCK across tissue subregions detected using a quantitative pathological analysis system; (**I**-**N**) 3D colocalization module for calculating the interaction rates among the three molecular pairs (HSPA8-GEMIN5, HSPA8-PanCK, and GEMIN5-PanCK) using a 100 × 100 μm grid scanning algorithm; (**O**) Spatial proximity analysis based on the HSPA8-positive regions (calculation distance: 25 μm), with super-resolution imaging technology used to measure the average spatial distance of the HSPA8-GEMIN5 molecular pairs; (**P**) Spatial proximity analysis based on the PanCK-positive regions (calculation distance: 25 μm), which were used to quantify the distance parameters of the PanCK-HSPA8/GEMIN5 complexes

In order to evaluate the clinical significance of the HSPA8-GEMIN5 interaction in CRC pathogenesis, a high-density tissue microarray (TMA) was constructed using 80 paired human CRC/adjacent normal tissue samples (Supplementary Fig. S12). The pathological relevance of these findings was elucidated in the subsequent multiplex immunofluorescence (IF) combined with spatial phenotyping analysis. Representative spatial expression patterns of HSPA8, GEMIN5, and the epithelial marker PanCK at different magnifications were shown in Fig. 7A-B. Compared to their adjacent normal counterparts, CRC tissues had significantly increased expressions of HSPA8 and GEMIN5, as revealed in quantitative multiplex IF (Fig. 7C-F), with synchronous overexpression patterns noted in the tumor cores. PanCK was similarly enriched in the tumor regions (Fig. 7G-H), suggesting the cooperative involvement of HSPA8, GEMIN5, and PanCK in malignant proliferation. Spatial interaction analysis revealed significantly greater HSPA8-GEMIN5 colocalization in the tumor regions compared to that in the adjacent or stromal regions (Fig. 7I-J), indicating the formation of functional “chaperone-RNA processing” complexes. The concurrent intensification of HSPA8-PanCK and GEMIN5-PanCK colocalizations within tumors (Fig. 7K-N) confirmed the selective enrichment of HSPA8-GEMIN5 complexes in proliferating epithelial cells, providing a rationale for the selectivity of this targeted therapy. Super-resolution spatial imaging further revealed a 2 μm reduction in the average intermolecular distance between HSPA8 and GEMIN5 in the tumor tissues (Fig. 7O), which was consistent with nanoscale functional complex formation. Additionally, a 4 μm decrease in the distance between PanCK-positive regions and the HSPA8-GEMIN5 complexes in tumors (Fig. 7P) indicated that nanodomain reorganization supported prosurvival clusters during malignant progression. These topological features established a direct basis for spatially selective anticancer drug development.

### DSHK induces the AS of RP-encoding genes through the functional impairment of GEMIN5

**Fig. 8:**
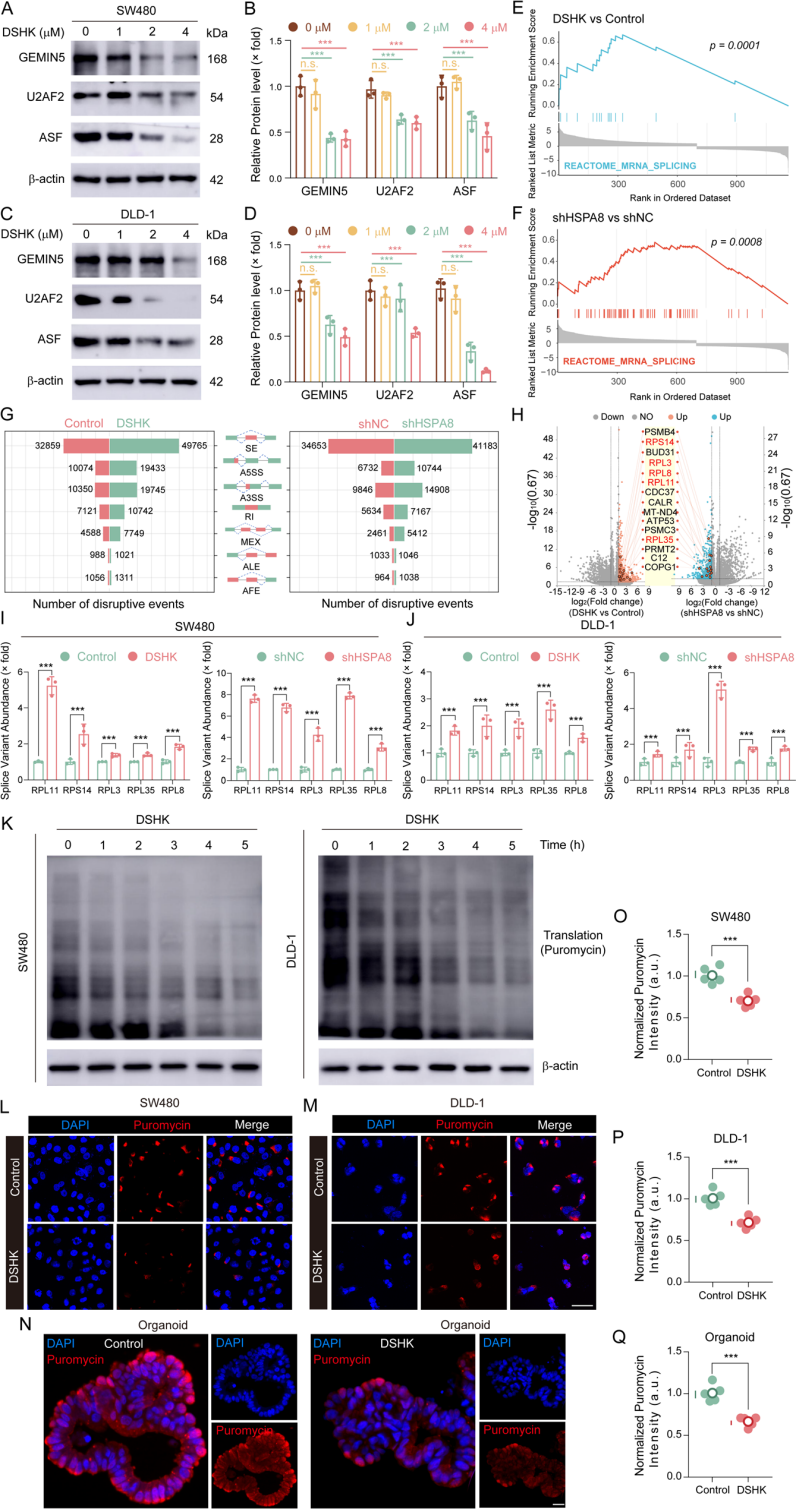
DSHK induces the AS of RP-encoding genes through GEMIN5 functional impairment. **A**-**D** Western blot quantification of GEMIN5 and splicing factor U2AF2/ASF protein expressions in SW480/DLD-1 cells after treatment with gradient DSHK (24 h); (**E**-**F**) GSEA pathway enrichment analysis of the DLD-1 cells treated with DSHK (4 µM, 24 h) or HSPA8-knockdown using whole-transcriptome sequencing (Illumina NovaSeq 6000); (**G**) Quantification of seven alternative splicing event frequencies using the rMATS v4.1.2 algorithm: skipped exon (SE), A5SS, A3SS, retained intron (RI), mutually exclusive exons (MXE), alternative promoter (AP), alternative polyadenylation (APA); (**H**) differential splicing gene screening in DLD-1 cells under DSHK exposure (4 µM, 24 h) or shHSPA8 intervention (threshold |ΔPSI| > 0.3, FDR < 0.05), with the RP genes highlighted in red; (**I**-**J**) Validation of the AS isoform expression of RP genes (RPL11/RPS14/RPL3/RPL35/RPL8) through qPCR, performed using specific primers, in the SW480/DLD-1 cells under DSHK exposure (4 µM, 24 h) or shHSPA8 intervention; (**K**) Puromycin-mediated nascent chain pulse-labeling (10 µg/mL, 30 min) used to determine the protein synthesis rates in SW480/DLD-1 cells after different periods under DSHK treatment (4 µM); (**L**-**Q**) Spatial distribution analysis of nascent protein synthesis through IF in SW480/DLD-1 cells (DSHK 4 µM, 5 h) and organoids (DSHK 8 µM, 7 days), scale bar = 25 μm. Data presented are the mean ± SD of at least three independent experiments (^***^P < 0.001)

Considering the essential role of the molecular chaperone HSPA8 in preserving the conformational stability of its associated proteins [13, 14] and the specific disruption of the HSPA8-GEMIN5 interaction interface by DSHK, the protein homeostasis and functional integrity of GEMIN5 could be substantially undermined. Given the crucial involvement of GEMIN5 in regulating RNA metabolism, particularly in spliceosome assembly and pre-mRNA processing [15, 16], this study systematically elucidated the modulatory effects of DSHK on the GEMIN5-mediated RNA metabolic network. Western blotting results demonstrated that DSHK treatment significantly decreased the protein levels of GEMIN5 and its key interacting factors U2AF2 and ASF in SW480/DLD-1 cells (Fig. 8A-D), indicating compromised RNA spliceosome assembly. Transcriptome sequencing analysis revealed that both DSHK treatment and HSPA8 knockdown substantially increased the number of genome-wide abnormal AS events (Fig. 8E-F). Quantitative analysis conducted using the rMATS algorithm revealed elevated frequencies of all seven AS event types in DSHK-treated or HSPA8-knockdown cells (Fig. 8G). As shown in Fig. 8H, differential splicing screening revealed 15 significantly upregulated genes, including five RP-encoding genes (RPL11, RPS14, RPL3, RPL35, and RPL8). AS events in these ribosomal protein genes primarily involved alternative 3’ splice sites (A3SS) and alternative 5’ splice sites (A5SS). The qRT-PCR results confirmed that both DSHK treatment and HSPA8 knockdown significantly increased the expressions of different AS isoforms of these genes in the SW480/DLD-1 cells (Fig. 8I-J), demonstrating impaired splicing fidelity in RP-encoding genes. According to the hypothesis that RP dysfunction may disrupt protein synthesis, the effect of DSHK on protein production was investigated next. Puromycin-mediated nascent chain labeling assays revealed that DSHK treatment significantly suppressed the nascent protein synthesis rates in both SW480/DLD-1 cells and organoid models (Fig. 8K-Q). Collectively, these findings established that DSHK induces AS of RP-encoding genes through the functional impairment of GEMIN5, consequently reducing the protein synthesis rates.

### DSHK impedes functional ribosome biogenesis

**Fig. 9:**
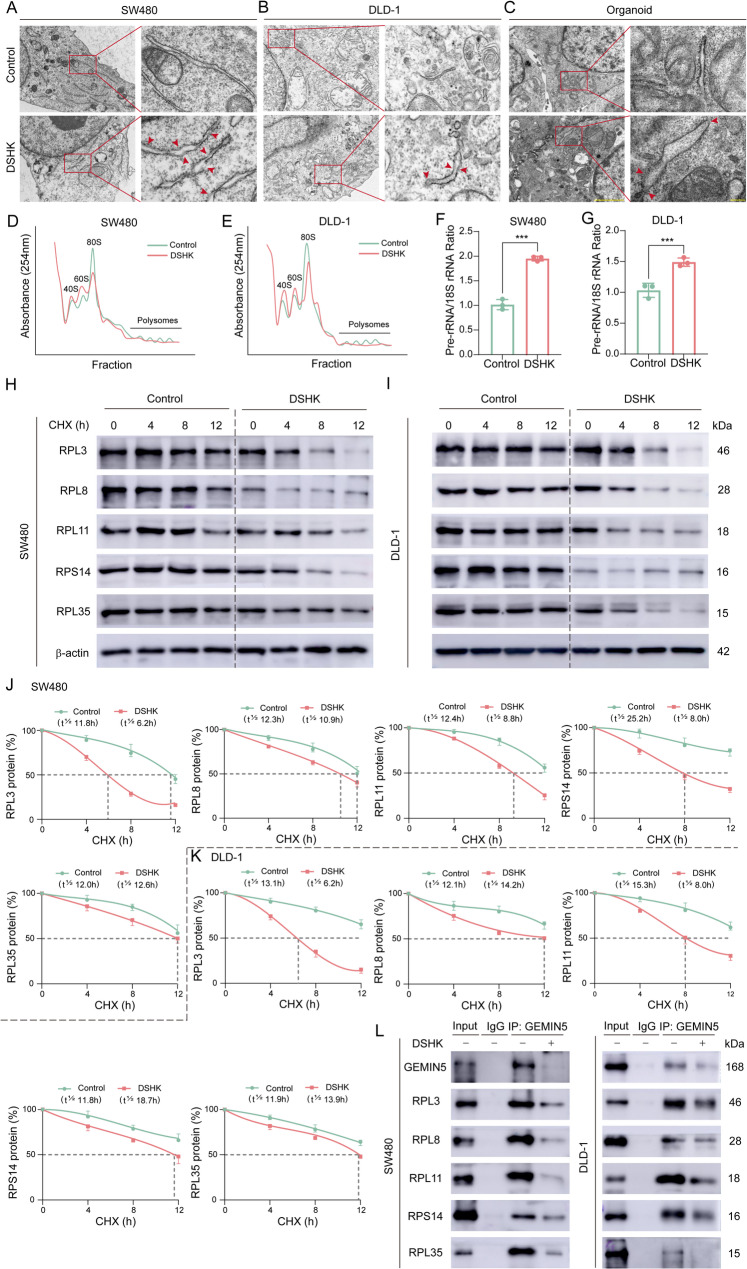
DSHK impairs functional ribosome biogenesis. **A**-**C** Transmission electron microscopy (JEM-1400Plus, 120 kV) image of the rough endoplasmic reticulum ultrastructure in DSHK-treated SW480/DLD-1 cells (4 µM, 5 h) and the organoids (8 µM, 7 days) following dual staining with uranyl acetate/lead citrate; (**D**-**E**) Sucrose density gradient centrifugation analysis (10%-50% gradient, Beckman SW41 Ti rotor, 40,000 rpm for 2 h) of the SW480/DLD-1 cells treated with DSHK (4 µM, 5 h); ribosomal subunit distribution (40 S/60S/80S) was detected based on the 254 nm absorbance spectrum; (**F**-**G**) Absolute quantitative qRT-PCR detection of pre-rRNA and mature rRNA (18 S) transcripts in the SW480/DLD-1 cells treated with DSHK (4 µM, 5 h); (**H**-**K**) Cycloheximide (CHX, 10 µg/mL) chase experiment combined with western blot analysis of ribosomal protein stability after DSHK intervention (4 µM, 5 h), with half-life calculated using the nonlinear regression fitting of grayscale decay curves; (**L**) Co-IP validation of GEMIN5-RP interaction intensity. The data presented are the mean ± SD of at least three independent experiments (^***^P < 0.001)

According to the AS of RP-encoding genes and the suppressed protein synthesis phenotypes noted in this study, it was hypothesized that DSHK might induce ribosome assembly impairment. Transmission electron microscopy ultrastructural imaging was, therefore, conducted, which revealed that DSHK-treated cells exhibited significant deribosomation at the rough endoplasmic reticulum membranes compared to controls, which was characterized by markedly reduced ribosomal density, disorganized spatial distribution (alternating sparse and dense regions), and the emergence of ribosomal-deficient bare membrane areas (indicated using arrows, Fig. 9A-C). In order to verify this ribosomal dysfunction, sucrose density gradient centrifugation was performed. As shown in Fig. 9D-E, compared to the control cells, DSHK-treated cells presented significantly attenuated 80 S mature ribosomal peaks and imbalanced 40 S/60S precursor subunit ratios. Absolute qRT-PCR quantification was performed next, which revealed substantially increased pre-rRNA/18S rRNA ratio after DSHK treatment due to impaired pre-rRNA processing maturation (Fig. 9F-G). Consequently, it was understood that DSHK induced global ribosomal dysfunction. Furthermore, DSHK treatment shortened the half-lives of the aberrantly spliced RPs (RPL3, RPL8, RPL11, RPS14, and RPL35) (Fig. 9H-K). Since GEMIN5 directly interacts with the RPs to maintain ribosomal function [8], Co-IP assays were performed, which revealed that DSHK weakened the interactions between GEMIN5 and RPs (RPL3, RPL8, RPL11, RPS14, and RPL35) in SW480/DLD-1 cells (Fig. 9L). Overall, it was understood that DSHK compromises functional ribosome biogenesis by disrupting the ribosomal subunit assembly cooperativity and structural integrity, ultimately inhibiting the efficiency of global protein biosynthesis.

### HSPA8 mediates DSHK-induced dysfunction of translation initiation

**Fig. 10:**
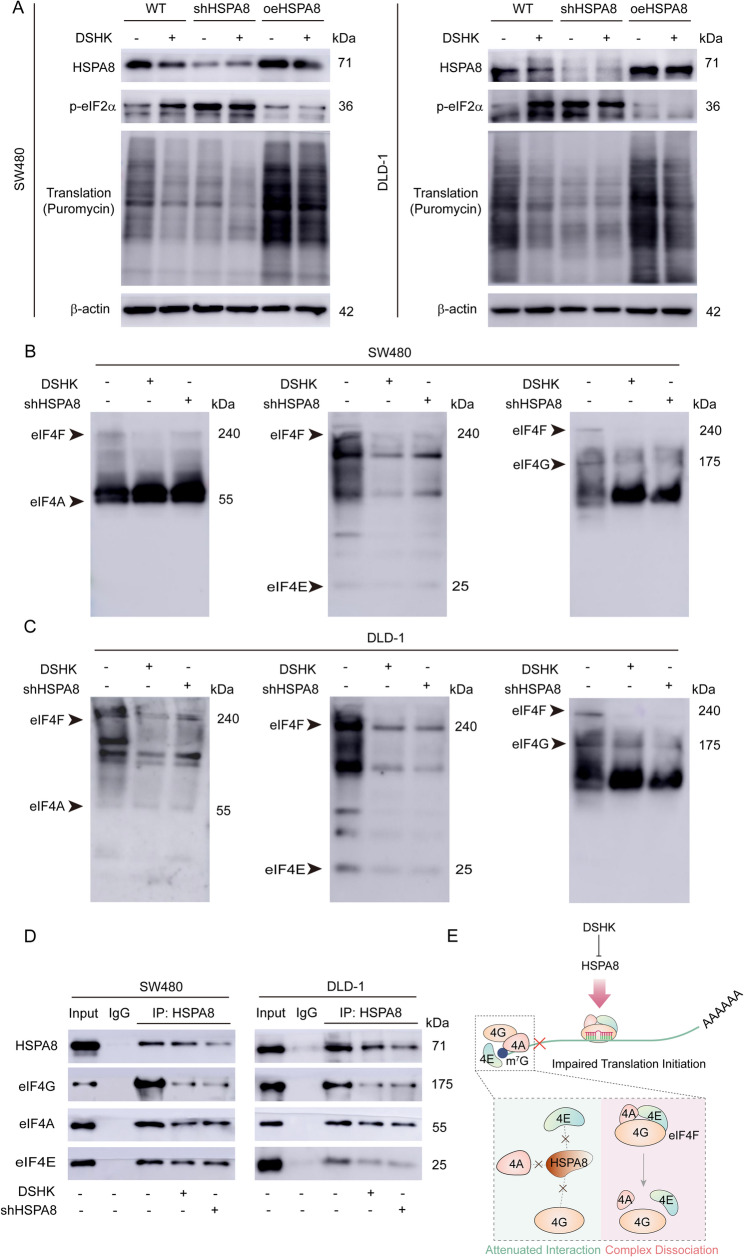
HSPA8 mediates DSHK-induced dysfunction of translation initiation. **A** In the SW480/DLD-1 cells treated with DSHK (4 µM, 5 h), HSPA8 knockdown, or HSPA8 overexpression, translation activity was detected using a puromycin incorporation assay (10 µg/mL, 30 min), and p-eIF2α levels were quantified using western blotting to evaluate translation initiation activity; (**B**-**C**) In the SW480/DLD-1 cells treated with DSHK (4 µM, 5 h) or HSPA8 knockdown, the assembly status of the eIF4F complex (molecular weight ≈ 240 kDa) was analyzed using native polyacrylamide gel electrophoresis (native PAGE, 4%-20% gradient gel); (**D**) Quantitative Co-IP assays conducted for the SW480/DLD-1 cells treated with DSHK (4 µM, 5 h) or HSPA8 knockdown validated the interaction strength between HSPA8 and the translation initiation factors (eIF4A/4G/4E); (**E**) Schematic diagram of the mechanism through which DSHK induces the dysfunction of protein synthesis translation initiation. The data presented are the mean ± SD of at least three independent experiments

Previous studies reported that HSPA8 promotes the assembly of the eukaryotic initiation factor-4 F (eIF4F) complex (comprising eIF4A, eIF4E, and eIF4G) by stabilizing the tertiary structures of translation initiation factors [6, 17]. Therefore, whether DSHK suppresses protein synthesis by disrupting translation initiation was investigated. As shown in Fig. 10A, HSPA8 knockdown reduced the translation levels while increasing the phosphorylation of eukaryotic initiation factor-2α (p-eIF2α), a marker of initiation blockade, in SW480/DLD-1 cells. Conversely, HSPA8 overexpression increased the translation levels and decreased the p-eIF2α levels. Similarly, DSHK treatment reduced translation and increased p-eIF2α, and these effects were attenuated by modulating the HSPA8 expression (Fig. 10A). Thus, it was understood that DSHK specifically targets HSPA8 to impair translation initiation. Native PAGE analysis revealed that both HSPA8 knockdown and DSHK treatment induced the dissociation of the eIF4F complex (eIF4A/eIF4G/eIF4E) in SW480/DLD-1 cells (Fig. 10B-C). Co-IP assays further revealed the weakened interactions between HSPA8 and eIF4A/eIF4G/eIF4E after HSPA8 depletion or DSHK treatment (Fig. 10D). Overall, DSHK destabilizes the HSPA8-mediated initiation factor interactions and eIF4F complex integrity, thereby inducing translational initiation dysfunction (Fig. 10E).

## Discussion

The high heterogeneity of CRC significantly limits the clinical translation efficiency of targeted therapies [18, 19]. The currently used targeted agents face the dual challenge of limited target coverage and compensatory pathway activation [20]. In this context, natural small-molecule compounds serve as an indispensable strategic approach to overcome these limitations encountered in targeted therapy owing to their unique structural diversity and multitarget synergistic regulation capabilities [21–23]. The in-depth mechanistic elucidation of natural small-molecule compounds is key to deciphering the biological essence of drug-target interactions and may also serve as a critical breakthrough for developing efficient and low-toxicity anticancer agents [24, 25]. DSHK is a natural small-molecule compound that has demonstrated promising antitumor potential across multiple preclinical cancer models [26, 27]. This study further confirmed the selective inhibitory efficacy of DSHK against high-incidence malignancies, particularly CRC. Nevertheless, the precise molecular targets and downstream regulatory networks of DSHK remain to be fully characterized, and this mechanistic knowledge gap currently impedes the clinical translation of DSHK.

The present study demonstrated that DSHK specifically disrupts the interaction of HSPA8-GEMIN5, resulting in the inactivation of HSPA8’s molecular chaperone function [13, 14], which impairs the protein homeostasis and functional integrity of GEMIN5 and significantly increases the AS frequency of RP-encoding genes, thereby inducing ribosome assembly defects and translation initiation impairment. Compared to shikonin (SHK), which acts through the NEMO/IKKβ pathway [26], its structurally optimized derivative DSHK exhibits a unique HSPA8-dependent mechanism with potential advantages in terms of reduced systemic toxicity. Molecular validation in this study confirmed HSPA8 as the direct target of the anti-CRC action of DSHK, with the ASP10 residue constituting the critical binding site in this interaction. Functional studies revealed that DSHK disrupts HSPA8-GEMIN5 complex stability, leading to a loss of RP splicing fidelity, concurrently inducing translation initiation dysfunction. Across three preclinical CRC models (cell lines/PDOs/xenografts), DSHK significantly suppressed tumor growth without causing systemic toxicity. Spatial pathomics analysis confirmed the nanoscale colocalization of HSPA8 and GEMIN5 within the tumor epithelium, providing theoretical support for precision targeting strategies and a mechanistic basis for the DSHK tumor-selective toxicity profile.

This study advances the current research paradigms across three dimensions. First, it redefines the functionality of molecular chaperones beyond conventional understanding. While the classical theory emphasizes the role of HSPA8 in maintaining protein folding homeostasis during stress responses [28], the integrated multiomics and functional validation data of this study revealed that HSPA8 dynamically regulates the splicing fidelity of RP-encoding genes by stabilizing the half-life of GEMIN5 protein. These findings indicate that the HSP70 family may constitute a cooperative regulatory network bridging RNA metabolism and ribosome assembly. These findings critically supplement the traditional HSP70 models by (i) elucidating chaperone functional plasticity under basal conditions and (ii) revealing a trans-dimensional regulatory mechanism mediated by RNA-binding protein-dependent splicing-translation coupling. This “chaperone-RNA binding protein” functional alliance could represent a core regulatory hub for ribosome biogenesis and may provide novel perspectives for understanding the precise control of protein synthesis networks in non-stressed conditions, potentially explaining the basis of tumor proteome diversity [8, 29]. Second, a novel paradigm in ribosome regulation theory was established in this study. Unlike prior studies that were focused on copy number variations or expression dysregulation of RP genes [30], this study proposed the concept of “splicing-dependent ribosomal stasis”, which states that aberrantly spliced RPs induce translational efficiency decay through subunit ratio imbalance and pre-rRNA processing blockade. This concept not only provides a novel mechanistic explanation for the widely observed ribosomal stasis in tumors but also positions splicing error-mediated ribosome inactivation as a key regulatory mechanism independent of the classical p53-nucleolar stress pathway [31, 32]. This cascade of RNA processing defects, demonstrated to directly drive translational dysfunction, may open novel avenues for understanding the feedback loops between protein synthesis and genomic instability in malignancies [33, 34]. Third, this study pioneers in reporting a novel natural small-molecule compound intervention strategy, positioning DSHK as the first reported natural small-molecule compound that targets the “splicing-translation coupling” axis, resulting in the inhibition of protein synthesis through the specific dissociation of the HSPA8-GEMIN5 complex. Compared to the conventional ribosome-targeting agents (e.g., CX-5461’s single-mechanism inhibition of RNA Pol I [4, 5]), DSHK offers a dual-pathway (splicing fidelity loss and translation initiation impairment) intervention, which may effectively circumvent therapeutic resistance. This strategy provides novel insights for overcoming the compensatory hyperactivation of ribosome biogenesis in solid tumors, and its protein-protein interaction interface-targeting modality lays the foundation for developing low-toxicity allosteric inhibitors [35, 36]. Notably, in the CRC organoid model featuring a three-dimensional barrier, DSHK still demonstrates significant anti-tumor activity, indicating that DSHK exhibits strong capabilities for tumor tissue penetration and accumulation.​.

While this study systematically elucidates the anti-CRC mechanism of action of DSHK, which targets the HSPA8-GEMIN5 complex to regulate “splicing-translation coupling”, several critical aspects warrant further exploration. As shown in Fig. 4L, HSPA8 ablation abrogated the antitumor efficacy of DSHK in vivo, indicating that the anti-CRC effects of DSHK depended on HSPA8. However, the antitumor effect of HSPA8 knockdown alone was superior to that of DSHK alone. The primary reasons may be as follows: First, given the critical role of HSPA8 in maintaining cancer cell growth, survival, and stress response, shRNA-mediated gene knockdown can achieve sustained, stable, and nearly complete inhibition of HSPA8 function. In contrast, DSHK, as a natural small-molecule inhibitor, is limited by pharmacokinetic factors in vivo, potentially failing to achieve sustained and complete inhibition of HSPA8 function within the tumor site. Second, besides HSPA8, DSHK may have other secondary targets, and potential off-target effects could partially influence its antitumor activity, thereby making its monotherapeutic efficacy weaker than that of specific target gene knockdown. At the molecular mechanism level, the basis for the specific recognition of RP pre-mRNAs by the HSPA8-GEMIN5 complex remains to be fully characterized. Future studies could employ cryoelectron microscopy to resolve the dynamic interaction conformations between the HSPA8-GEMIN5 complex and pre-mRNAs, revealing their spatial recognition codes. Additionally, the spatiotemporal dynamics of the disruption of ribosomal quality control by the aberrantly spliced RP isoforms have to be precisely defined. Single-particle tracking technology could enable the real-time monitoring of ribosome assembly processes, thereby providing kinetic evidence for “splicing-dependent proteostasis imbalance”. In translational applications, the in vivo targeting efficiency of DSHK may be limited by the permeability of the tumor microenvironment. The development of pH-responsive nanocarriers targeting HSPA8 could increase drug accumulation in tumor tissues. Leveraging the nanoscale colocalization signature of HSPA8 and GEMIN5 in the tumor epithelia, spatially resolved multiomics-guided patient stratification models could be constructed to facilitate the precise identification of the therapeutic benefit subgroups. These research directions would provide pivotal support for refining the theoretical frameworks and advancing clinical translation.

According to the findings of this study, two novel CRC therapeutic strategies with significant translational potential are proposed. First, novel precision therapeutic approaches have been developed, and by targeting the structural characteristics of the HSPA8-GEMIN5 interaction interface, allosteric inhibitors can be designed to selectively disrupt the stability of the HSPA8-GEMIN5 complex. This strategy may circumvent the systemic toxicity associated with the traditional HSP70 inhibitors used for targeting the ATPase domain [37]. Additionally, by integrating the AS profiles of the RP-encoding genes, patient subgroups with “splicing vulnerability” in CRC can be screened to achieve optimized precision application of DSHK. Second, innovative drug development paradigms are needed, and a three-dimensional evaluation system guided by organoid models has to be developed, which would integrate the following three key dimensions: drug sensitivity (assessed using PDO models), mechanistic interactions (validated using the SPIDER technology for target engagement), and spatial pathological analysis (conducted using tissue microarrays). This approach would provide a novel “activity-mechanism-clinical validation” system that can simultaneously achieve the standard for natural products, thereby enhancing the reliability of clinical translation decisions. These explorations are expected to pioneer novel approaches to precision CRC therapy and the development of anticancer natural products.

## Conclusions

Chemical biology target identification, functional interaction validation, and spatial pathomics analysis have been integrated in this study, elucidating a novel anti-CRC mechanism involving the natural small-molecule compound DSHK. DSHK specifically targets the HSPA8-GEMIN5 protein interaction interface, thereby disrupting the splicing-translation coupling network and inducing proteostasis imbalance. This discovery expands the functional paradigm of the molecular chaperone activity of HSPA8 from mere protein quality control to RNA metabolism regulation while establishing a “dual-pathway intervention” strategy for targeting both splicing and translation processes for solid tumor therapy. These findings would substantially advance the development of precision medicine for treating CRC.

## Supplementary Information

Supplementary Material 1.

## Data Availability

The RNA sequencing data generated in this study have been deposited in the National Center for Biotechnology Information (NCBI) under BioProject accessions PRJNA1289263 and PRJNA1288703. All other research data described herein are available from the corresponding author upon reasonable request.

## Abbreviations

A3SS: Alternative 3’ splice site
A5SS: Alternative 5’ splice site
AP: Alternative promoter
APA: Alternative polyadenylation
ACADVL: Acyl-CoA dehydrogenase, very long chain
Alanine: Alanine
AS: Alternative splicing
ASF: Serine/arginine-rich splicing factor 1
ATP: Adenosine triphosphate
CCK-8: Cell Counting Kit-8
CDX: Cell line-derived xenograft
CETSA: Cellular thermal shift assay
CHTOP: Chromatin target of PRMT1
CHX: Cycloheximide
Co-IP: Co-immunoprecipitation
CRC: Colorectal cancer
DSHK: Deoxyshikonin
E-cadherin: Epithelial cadherin
EdU: 5-Ethynyl-2’-deoxyuridine
eIF4A: Eukaryotic translation initiation factor 4 A
eIF4E: Eukaryotic translation initiation factor 4E
eIF4F: Eukaryotic translation initiation factor 4 F
eIF4G: Eukaryotic translation initiation factor 4G
GEMIN5: Gem Nuclear Organelle Associated Protein 5
H&E: Hematoxylin and eosin
H1-0: H1 histone family member 0
HSPA8: Heat shock protein family A member 8
HSPs: Heat shock proteins
IHC: Immunohistochemistry
IKKβ: Inhibitor of nuclear factor kappa B kinase subunit beta
MUC2: Mucin 2
MXE: Mutually exclusive exons
NCBI: National Center for Biotechnology Information
NEMO: NF-kappa-B essential modulator
NF-κB: Nuclear factor kappa B
OE: Overexpression
PanCK: Pan-cytokeratin
PCNA: Proliferating cell nuclear antigen
PDO: Patient-derived organoid
PDOX: Patient-derived organoid xenograft
p-eIF2α: Phosphorylated eukaryotic translation initiation factor 2 subunit alpha
PKM2: Pyruvate kinase M2
qRT-PCR: Quantitative reverse transcription polymerase chain reaction
Rg: Radius of gyration
RI: Retained intron
RMSD: Root mean square deviation
RMSF: Root mean square fluctuation
RPL11: Ribosomal protein L11
RPL3: Ribosomal protein L3
RPL35: Ribosomal protein L35
RPL8: Ribosomal protein L8
RPS14: Ribosomal protein S14
RPs: Ribosomal proteins
ROS: Reactive oxygen species
SASA: Solvent accessible surface area
SE: Skipped exon
sh-: Short hairpin RNA prefix
