# Supplementary material for: Disruption of HSPA8-GEMIN5 interaction suppresses colorectal cancer by impaired splicing-translation coupling-mediated proteostasis imbalance
Source: J Exp Clin Cancer Res. 2026 Jan 16;45:47. doi: 10.1186/s13046-026-03645-2 (PMC12892724; doi:10.1186/s13046-026-03645-2)
Supplement: Supplementary file 1 — Supplementary Material 1. [file 13046_2026_3645_MOESM1_ESM.docx]

Supplementary Material

**Disruption of HSPA8-GEMIN5 interaction suppresses colorectal cancer by impaired splicing-translation coupling-mediated proteostasis imbalance**

This WORD file includes:

Supplementary Figures S1-12

Materials and Methods

Supplementary tables for methods

**Supplementary Figure S1**

**
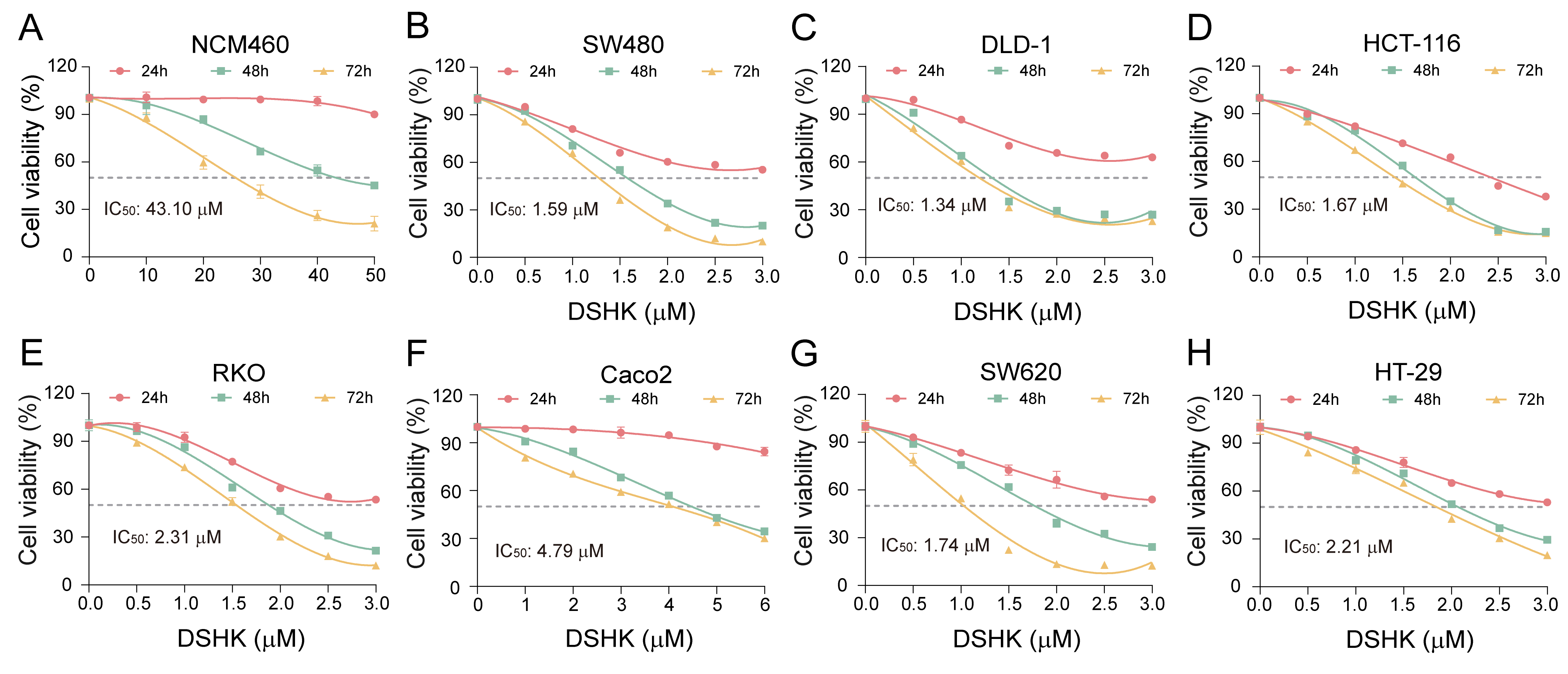
**

**Supplementary Figure S1. Effects of DSHK on the cell viability of normal human colon epithelial cells and human CRC cells.** (A) After treatment with gradient concentrations of DSHK for 24/48/72 h, the cell viability of human normal colon epithelial cell line NCM460 was measured using the CCK-8 method to determine the IC_50_ value. The IC_50_ value at 48 h of exposure was calculated. (B-H) After treatment with gradient concentrations of DSHK for 24/48/72 h, the cell viability was assessed based on seven human colorectal cancer cell lines (HCT-116, RKO, SW480, DLD-1, Caco-2, SW620, HT-29), and the IC_50_ values at 48 h of exposure were calculated. Data were presented as the mean ± SD of at least three independent experiments.

**Supplementary Figure S2**

**
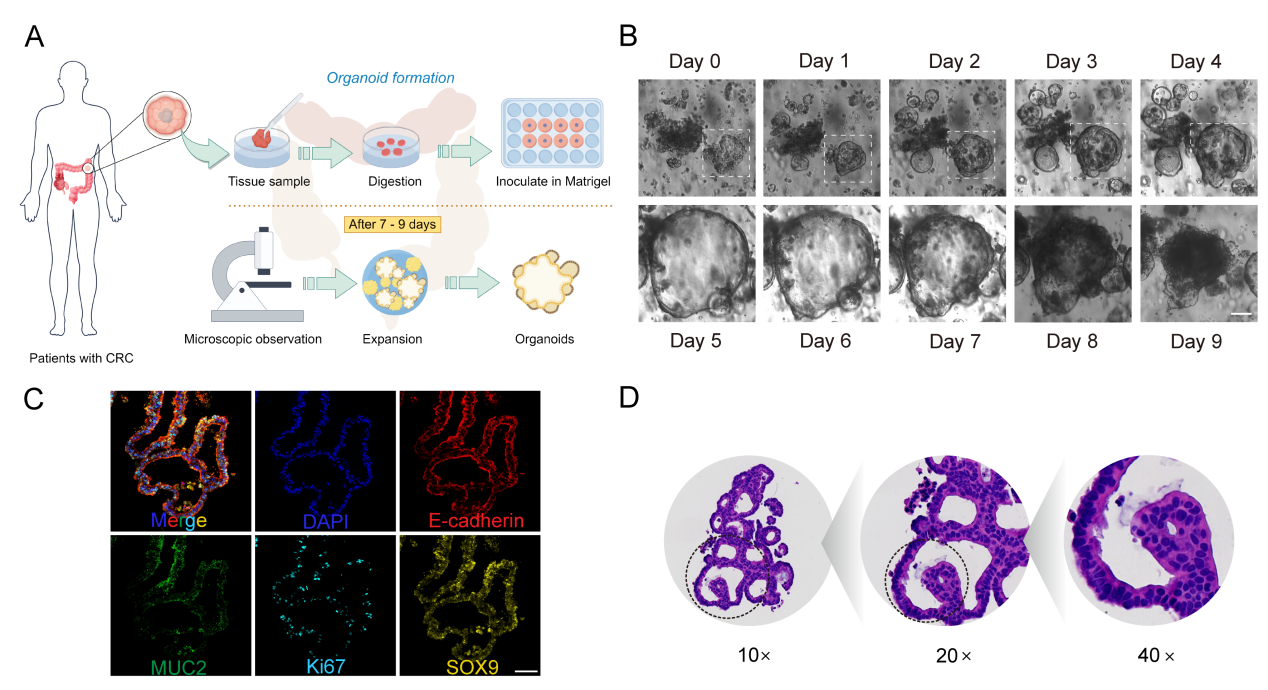
**

**Supplementary Figure S2.** **Establishment and characterization of patient-derived organoid (PDO) models.** (A) Schematic of PDO generation: CRC tissues underwent enzymatic digestion followed by embedding in Matrigel for 7-9 days to form mature organoids; (B) Bright-field microscopy dynamically monitored PDO growth (Scale bar = 200 μm), documenting morphological evolution; (C) Multiplex immunofluorescence (IF) validated core PDO markers: E-cadherin (intercellular adhesion; red), MUC2 (mucin secretion; green), Ki67 (proliferative activity; cyan), SOX9 (stemness; yellow) (Scale bar = 50 μm); (D) H&E staining analyzed PDO histological architecture, revealing glandular structures mirroring primary tumors.

**Supplementary Figure S3**

**
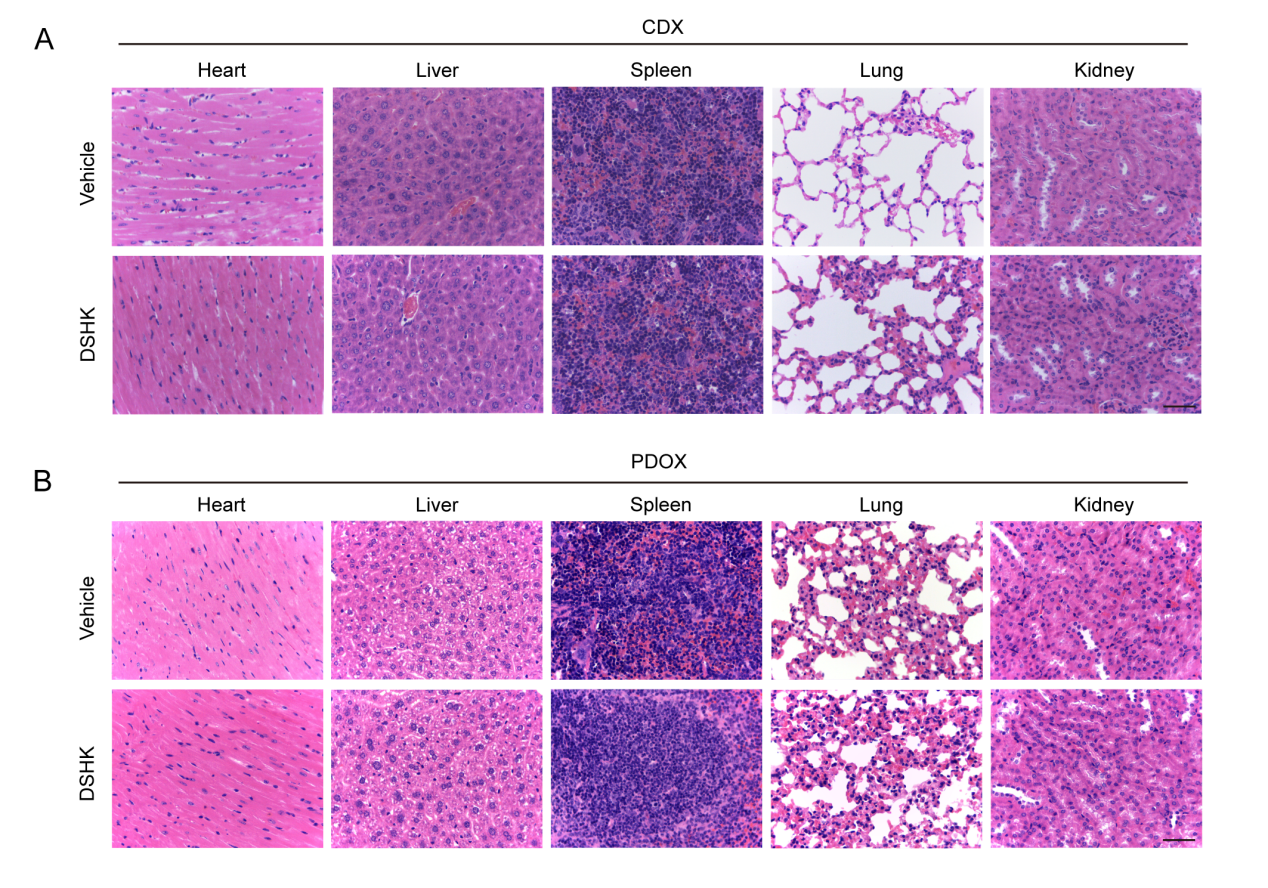
**

**Supplementary Figure S3. *In vivo* safety evaluation of DSHK.** (A-B) Systematic evaluation of histopathological features in mouse heart, lung, liver, spleen, and kidney tissues following DSHK intervention using H&E staining, analyzing in vivo toxicity responses in cell line-derived xenograft (CDX, n = 8) and patient-derived organoid xenograft (PDOX, n = 6) models. Scale bar = 25 μm.

**Supplementary Figure S4**

**
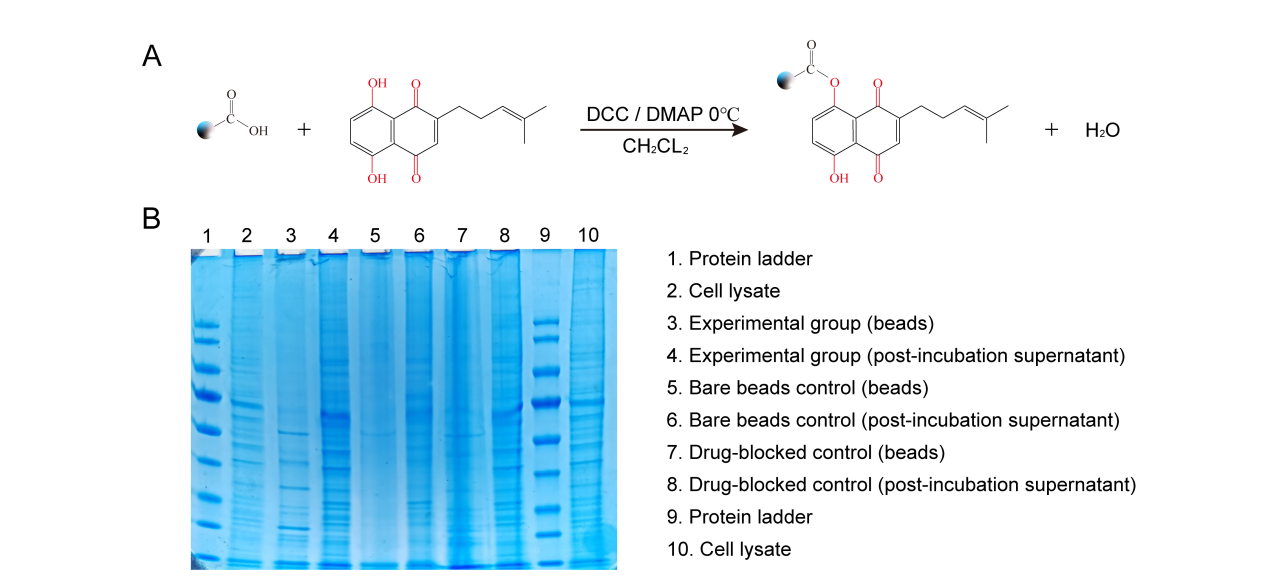
**

**Supplementary Figure S4. Direct target fishing for DSHK’s anti-CRC effect.** (A) Schematic illustrating the chemical proteomics-based target fishing principle for identifying direct targets of DSHK’s anti-CRC action: The active hydroxyl group of DSHK was captured via carboxyl microsphere esterification reaction; (B) Protein screening results derived from the target fishing strategy: SDS-PAGE gel electrophoresis (12% separating gel, 30 μL/lane loading volume) revealed differentially bound protein bands stained with Coomassie Brilliant Blue R250.

**Supplementary Figure S5**

**
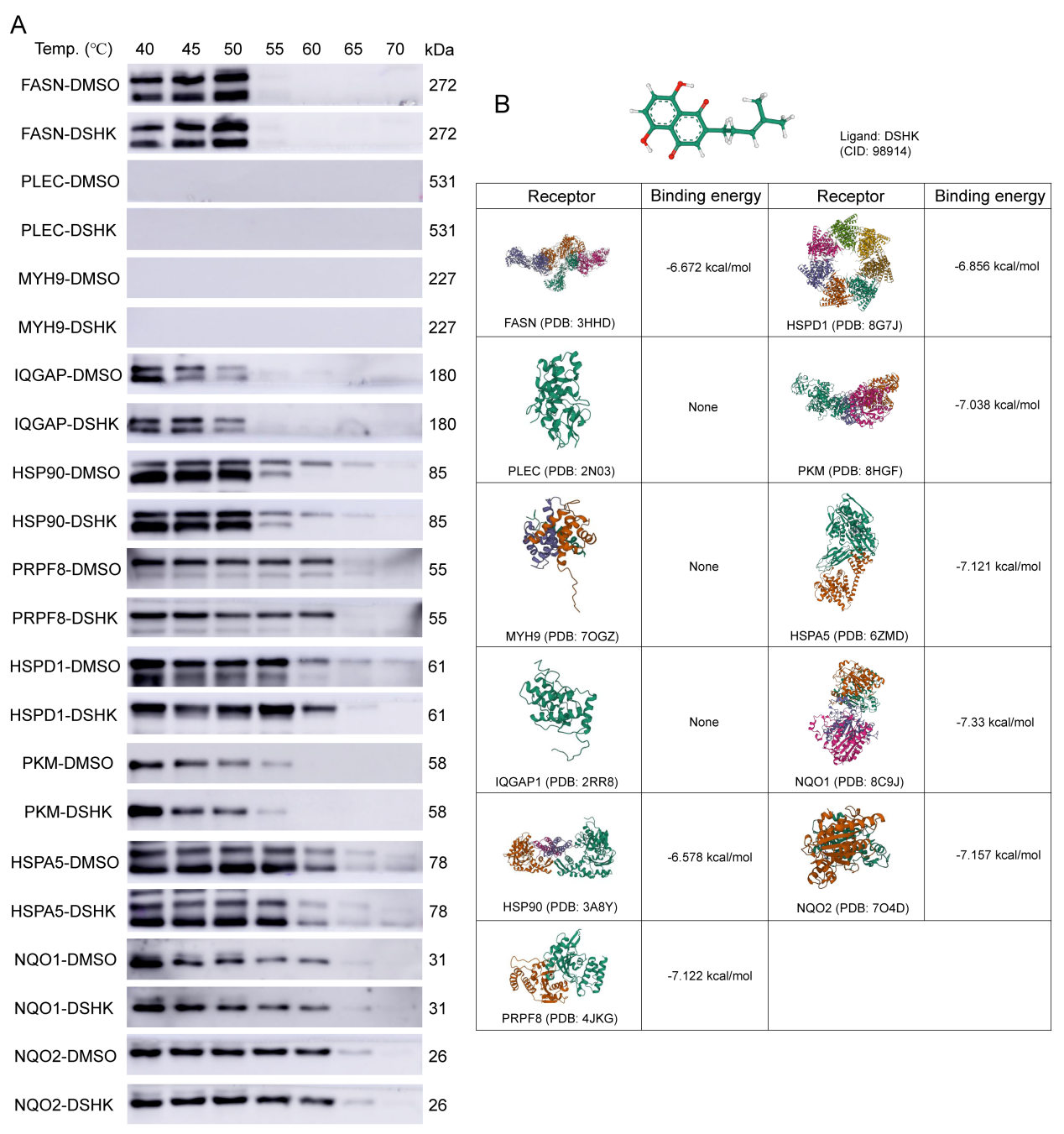
**

**Supplementary Figure S5. Preliminary validation of direct targets for DSHK’s anti-CRC effect.** (A-B) Functional validation studies of the top 12 high-confidence candidate targets identified through chemical proteomics fishing, employing cellular thermal shift assay (CETSA) and molecular docking scoring analyses.

**Supplementary Figure S6**

**
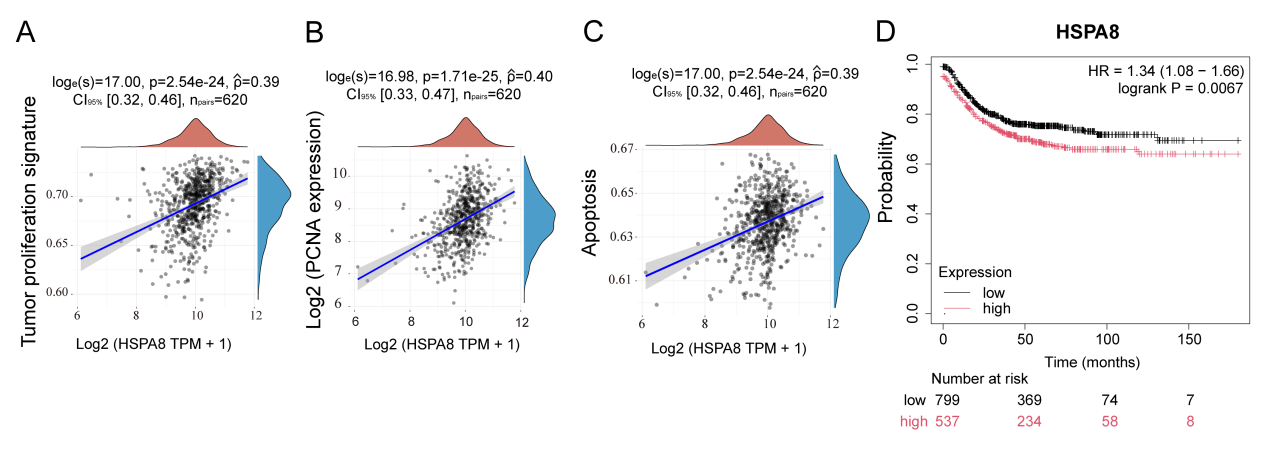
**

**Supplementary Figure S6. Correlation analysis of HSPA8 in CRC.** (A-C) Analysis of correlations between HSPA8 expression and tumor proliferation/apoptosis indices in the TCGA CRC cohort. (D) Replot the survival curve of HSPA8 using the Kaplan-Meier method. All data derived from the TCGA CRC database.

**Supplementary Figure S7**

**
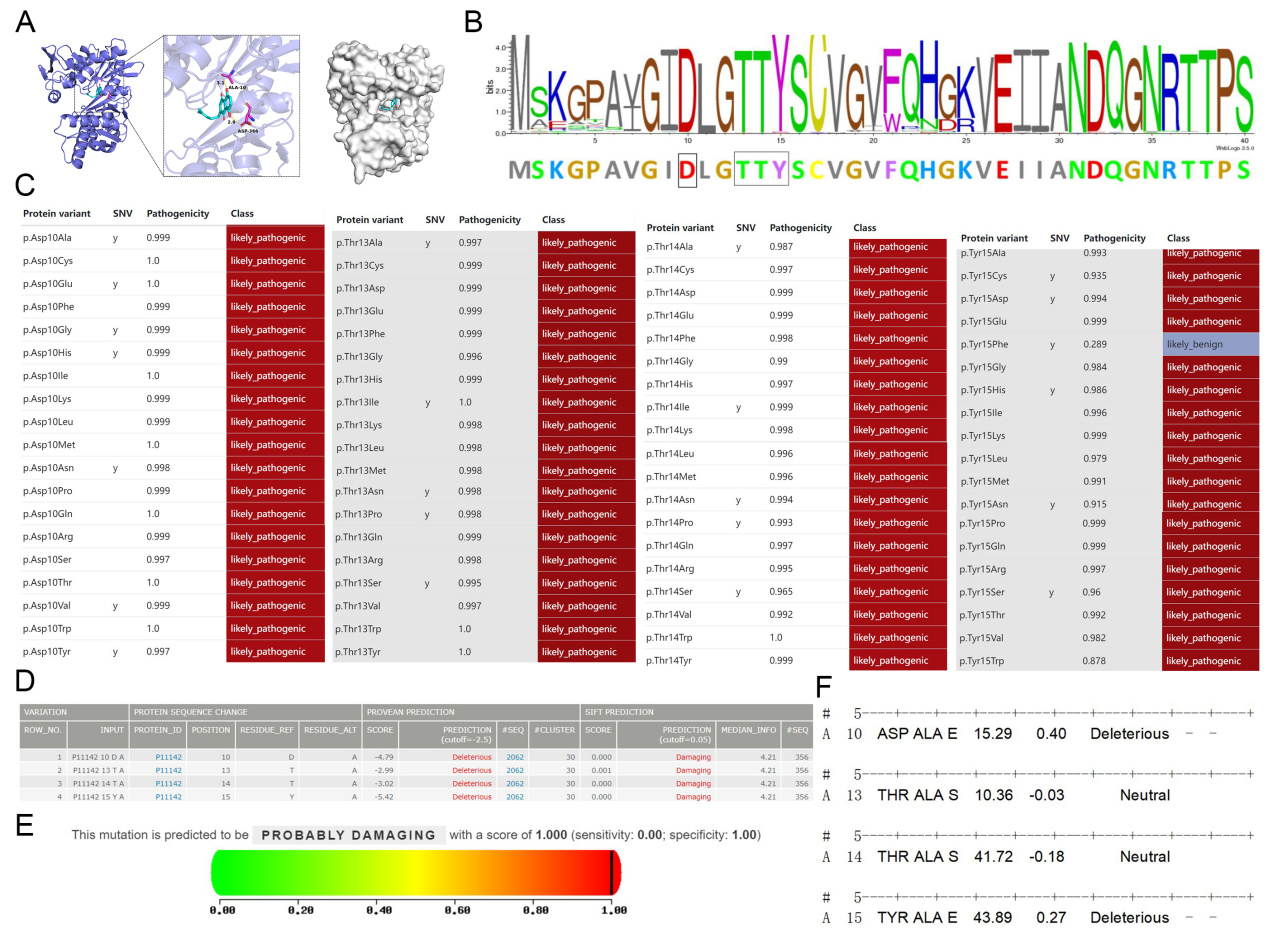
**

**Supplementary Figure S7. Functional characterization of key amino acid residues at the DSHK-HSPA8 binding interface.** (A) Molecular docking analysis of DSHK with the HSPA8 quadruple mutant (D10A/T13A/T14A/Y15A) revealed reduced binding affinity (ΔG = -6.9 kcal/mol) compared to wild-type (ΔG = -7.9 kcal/mol); (B-F) Integrated multi-algorithm assessment of evolutionary conservation for critical residues: Systematic analysis of ASP10/THR13/THR14/TYR15 conservation across vertebrate species using PolyPhen-2 v2.4.0 (pathogenicity probability of missense mutations), PROVEAN v1.1.3 (functional impact score), PoPMuSiC v3.1 (protein stability change), and VarSite (structural conservation index).

**Supplementary Figure S8**

**
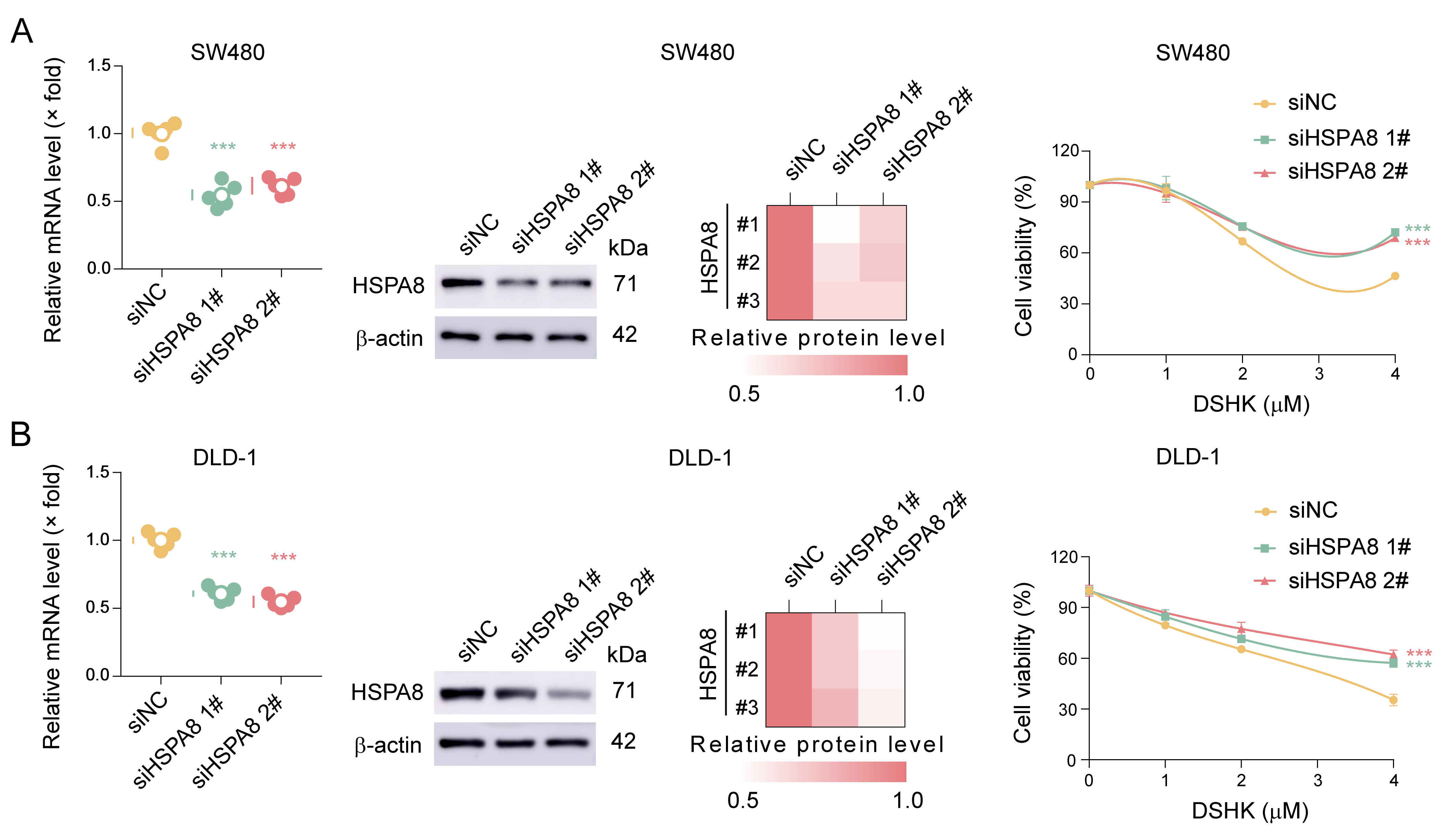
**

**Supplementary Figure S8**. **Impact of HSPA8 knockdown on DSHK’s anti-CRC efficacy.** (A-B) In SW480 and DLD-1 cells transfected with siHSPA8 1# or siHSPA8 2#: mRNA validation: qRT-PCR analysis of HSPA8 mRNA levels at 24 h post-transfection. Protein validation: Western blot analysis of HSPA8 protein expression at 48 h post-transfection. Pharmacodynamic impact: CCK-8 assay determination of cell viability under DSHK gradient concentration treatment (24 h exposure). Data were presented as the mean ± SD of at least three independent experiments (^***^*P* < 0.001).

**Supplementary Figure S9**

**
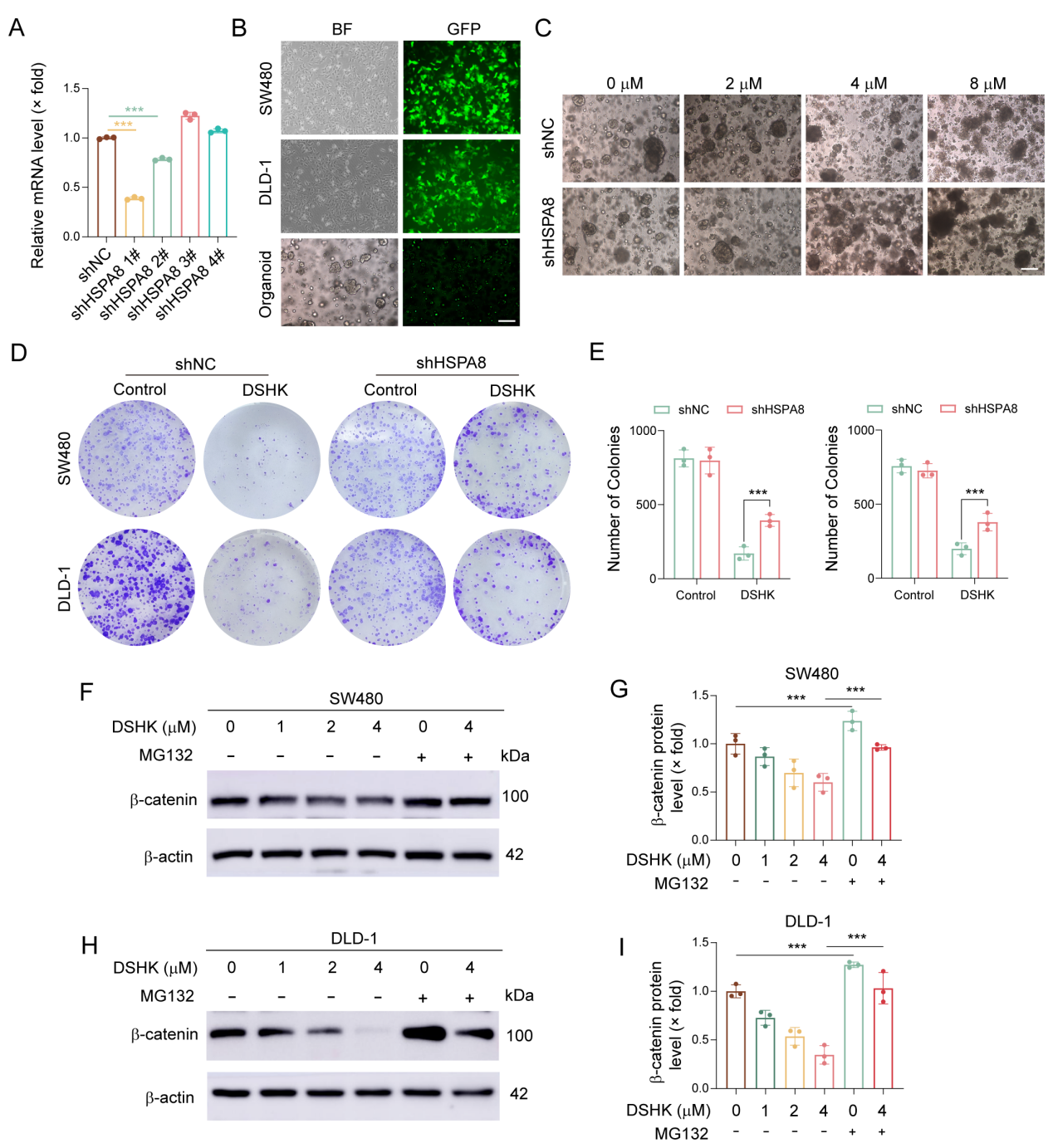
**

**Supplementary Figure S9. DSHK’s anti-CRC activity depends on HSPA8 expression.** (A) Screening of HSPA8 mRNA knockdown efficiency by four shRNA sequences in DLD-1 cells, validated via qRT-PCR; (B) GFP fluorescence intensity confirming >90% transduction efficiency in SW480/DLD-1 cells and organoids 72 h post-lentiviral infection (Scale bar = 100 μm); (C) Morphological impact of DSHK gradient concentrations on shNC/shHSPA8-infected CRC organoids after 7 day treatment (bright-field microscopy; Scale bar = 100 μm); (D-E) Quantitative analysis of colony formation inhibition in SW480/DLD-1 cells by HSPA8 knockdown combined with DSHK (4 μM) using colony formation assay (7 day culture). (F-I) Western blot analysis of the β-catenin ubiquitination degradation pathway in the DSHK-treated SW480/DLD-1 cells, with proteasome inhibitor MG132 pretreatment (4 μM, 4 h); Data were presented as the mean ± SD of at least three independent experiments (^***^*P* < 0.001).

**Supplementary Figure S10**

**
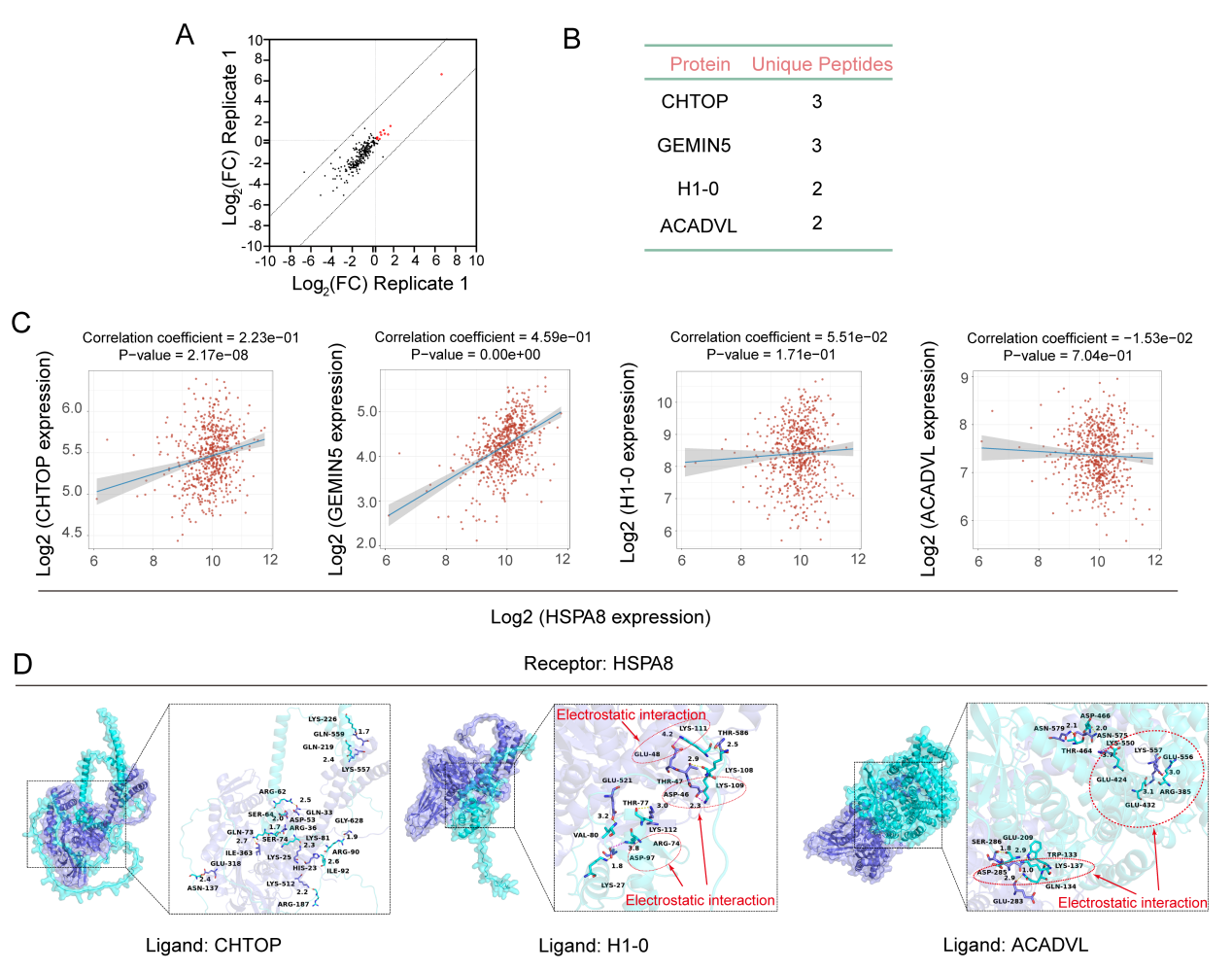
**

**Supplementary Figure S10. GEMIN5 as a novel functional interactor of HSPA8.** (A) Reproducibility analysis of HSPA8-interacting protein targets: Scatter plot showing strong positive correlation between protein signal intensities from two independent replicate experiments, with most data points distributed along the diagonal line indicating excellent experimental reproducibility; select outliers suggest specific targets requiring further validation. (B) Screening results of HSPA8-interacting proteins based on covalent binding enrichment. (C-D) Genomic correlation and molecular docking for four HSPA8 interactors: CHTOP (chromatin regulator), GEMIN5 (SMN complex core), H1-0 (linker histone), ACADVL (VLCAD enzyme).

**Supplementary Figure S11**

**
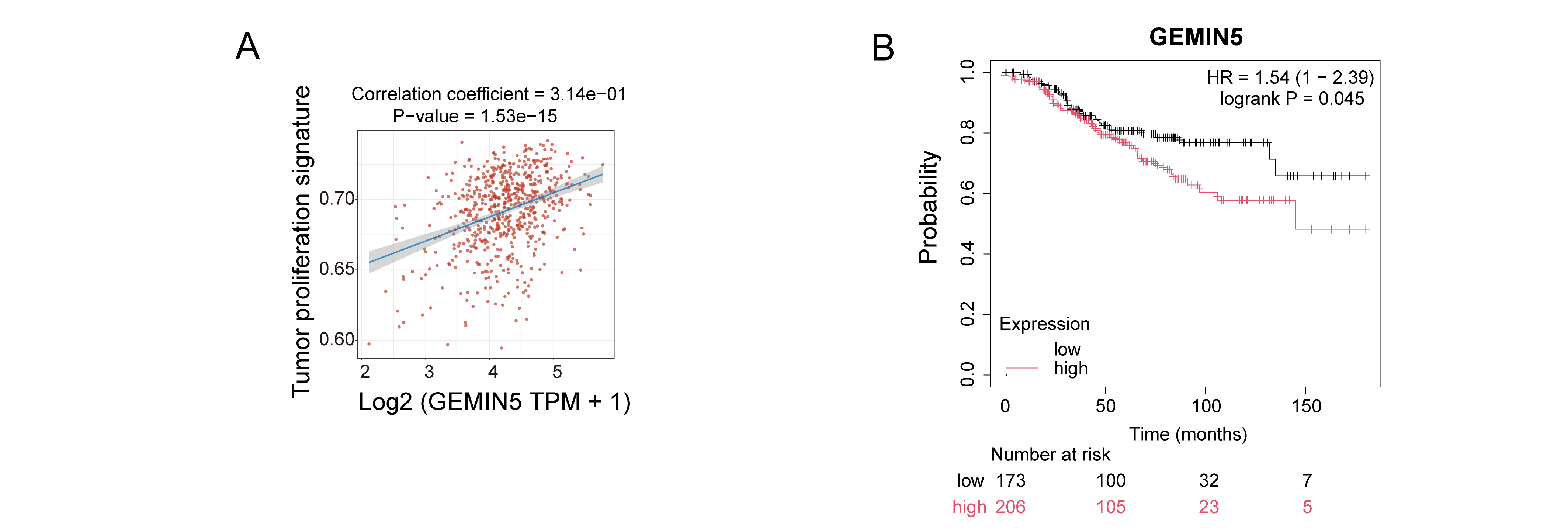
**

**Supplementary Figure S11. Correlation analysis of GEMIN5 in CRC.** (A) Analysis of the correlation between GEMIN5 expression and tumor proliferation indices in the TCGA CRC cohort; (B) Replot the survival curve of GEMIN5 using the Kaplan-Meier method. All data derived from the TCGA CRC database.

**Supplementary Figure S12**

**
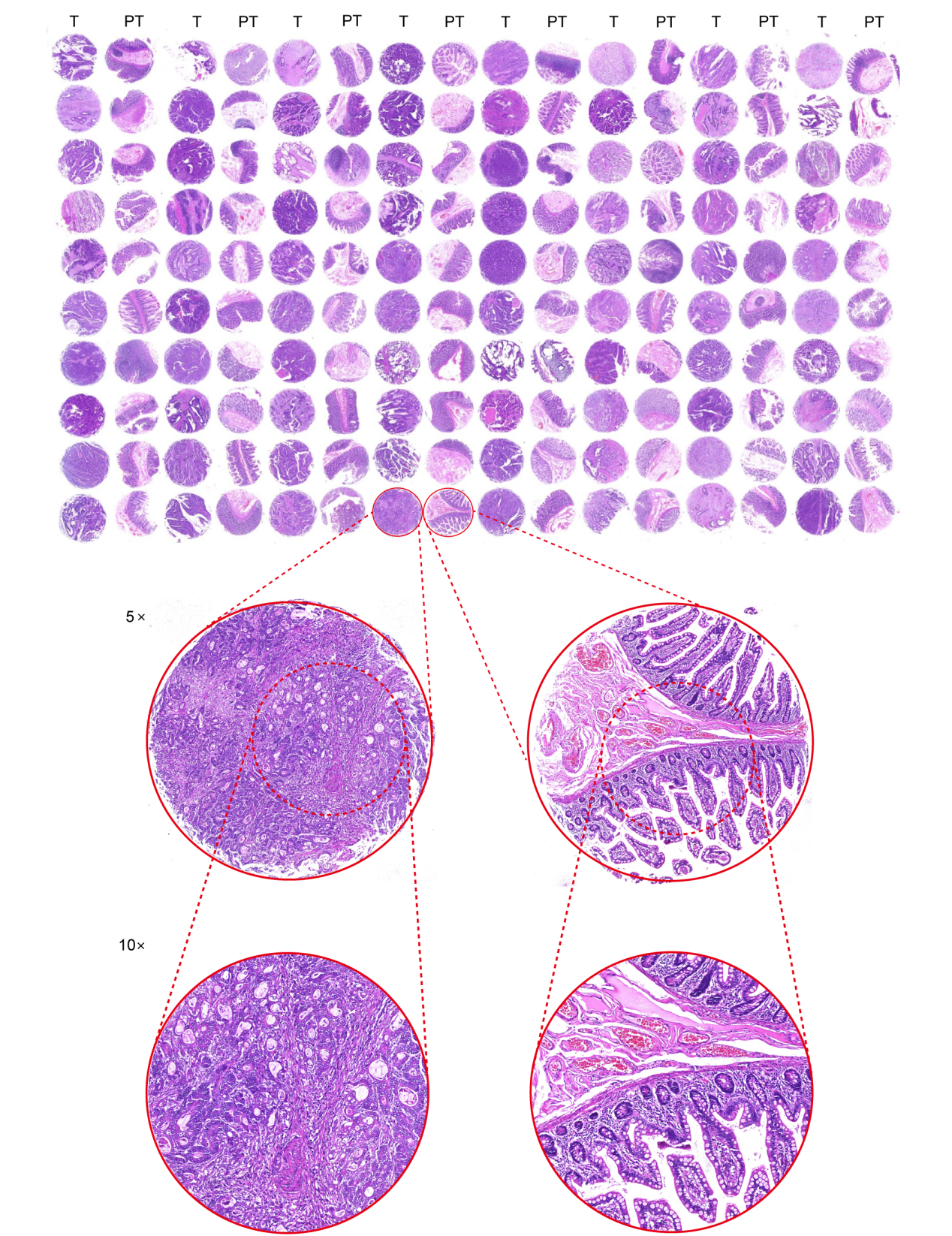
**

**Supplementary Figure S12. High-density tissue microarray (TMA) H&E staining of 80 paired human CRC tissue samples.** Representative multi-scale magnified views of tumor tissues (T) and matched peritumoral tissues (PT) from clinical cases are displayed.

**Materials and Methods**

**Cell and Organoid Culture Systems**

Human CRC cell lines SW480, DLD-1, HCT-116, RKO, Caco-2, SW620, HT-29, human normal colon epithelial cell line NCM460, and human embryonic kidney 293T (HEK293T) cells were procured from the Cell Resource Center of Peking Union Medical College (PUMCC, China) with authentication via STR profiling and mycoplasma contamination testing. Cells were cultured in medium supplemented with 10% fetal bovine serum (FBS) and 1% penicillin-streptomycin, maintained in RPMI-1640, while Caco-2 and HEK293T cells with high-glucose DMEM (Fuheng, Shanghai, China). Routine passaging occurred at 37°C/5% CO_2_ with 1:3-1:5 split ratios.

For human CRC organoid generation: Fresh tumor tissues were rinsed in PBS (1% penicillin-streptomycin), mechanically minced into <1 mm^3^ fragments, and enzymatically digested using collagenase IV (2 mg/mL) and dispase (1 mg/mL) with 37°C shaking for 30 min. Digested products were filtered through 70 μm strainers, centrifuged at 1000 rpm for 5 min, and resuspended in pre-chilled Matrigel (Corning, 356231, USA). Aliquots of 30 μL cell-Matrigel suspension were plated in pre-warmed 24 well plates, solidified at 37°C for 15 min, and overlaid with organoid medium (DaXiang Biotech, Beijing, China) containing: Noggin (50 ng/mL), R-spondin (100 ng/mL), EGF (50 ng/mL), Wnt3a (50 ng/mL), A83-01 (500 nM), SB202190 (3 μM), and B27 (1×). Medium was refreshed every 3 days, with organoids passaged via mechanical dissociation every 5-7 days. Experiments used stable passage 3-5 cultures with >90% viability.

**Establishment of Cell-Derived Xenograft (CDX) and Patient-Derived Organoid Xenograft (PDOX) Models**

Female BALB/c nude mice (SPF grade, 6-8 weeks old, 18 ± 2 g body weight) were purchased from Sibeifu (Beijing) Biotechnology Co., Ltd. and housed under SPF barrier conditions (temperature 22 ± 1°C, humidity 50 ± 10%, 12 h light/dark cycle) with ad libitum access to sterilized feed and purified water.

CDX Model: DLD-1 human CRC cells in logarithmic growth phase were digested with 0.25% trypsin and resuspended in PBS-Matrigel mixture (Mogengel-Bio, 082724, China; 1:1 v/v). Each mouse received a subcutaneous injection of 100 μL cell suspension (8 × 10^6^ cells) into the right flank. The tumor-bearing mice are monitored for survival status daily. Every 48 h, body weight and tumor volume are precisely measured (Tumor volume = Length × Width^2^ × 0.5). When the tumor volume reaches the ethical endpoint threshold (1500 mm^3^) or the body weight decreases by more than 20%, humane endpoint euthanasia is performed for tissue collection.

PDOX Model: CRC organoids (>95% viability, passage ≥3) were enzymatically dissociated with Accutase (37°C, 10 min) and resuspended in Matrigel-PBS mixture (1:1). Each mouse was subcutaneously injected with 100 μL suspension (5×10^5^ organoid cells) into the left flank. The remaining monitoring indicators and methods are consistent with those of the CDX model.

Terminal Procedures and Sample Collection: Mice were anesthetized via isoflurane inhalation and euthanized by cervical dislocation. Tumors were excised, rinsed in PBS, and weighed. Portions were flash-frozen in liquid nitrogen; remaining tissues were fixed in 4% paraformaldehyde (24 h) for paraffin embedding.

**Cell and Organoid Viability Assays**

Cell viability under DSHK treatment was evaluated using the CCK-8 assay. Logarithmically growing cells were seeded at 5×10^3^ cells/well in 96 well plates and pre-cultured for 12 h at 37°C/5% CO_2_ for adherence. Original medium was replaced with fresh medium containing DSHK gradient concentrations (solubilized in DMSO, final concentration ≤0.1%). After treatment, 10 μL CCK-8 reagent was added per well followed by 2 h incubation. Absorbance at 450 nm was measured using a microplate reader. Relative viability (%) was normalized to solvent control (0.1% DMSO) set as 100%. Experiments used 6 technical replicates per condition with triplicate biological repeats.

For CRC organoids, cytotoxic effects were assessed using a firefly luciferase-based ATP detection kit. Organoid spheroids (150-200 μm diameter) cultured for 5 days post-passaging were transferred to 96-well ultra-low attachment plates (10 uniform spheroids/well). After 24 h stabilization, medium was replaced with DSHK-containing complete medium. Post-treatment, pre-chilled ATP lysis buffer was added equally to each well and lysates were shaken at 37°C for 30 min. CellTiter-Glo 3D working solution was added for 20 min protected from light to induce luminescence. Luminescence intensity (RLU) was measured directly on a microplate reader without light excitation. ATP levels relative to 0.1% DMSO controls (100%) determined viability. Eight technical replicates were performed per group with triplicate independent experiments.

**ATP Content Quantification Method**

ATP levels were quantified using a firefly luciferase-based chemiluminescence detection kit (Meilunbio, MA0440, China). Post-DSHK treatment, cells were lysed with pre-chilled lysis buffer on ice, followed by centrifugation at 10,000 rpm (4°C, 5 min) to collect supernatants. ATP standards (0.5 mM) were serially diluted in lysis buffer to generate a 0.01-10 μM calibration curve. In black 96 well plates, 100 μL ATP detection reagent was added per well and equilibrated at room temperature for 5 min. Subsequently, 10 μL test samples or standards were added, with RLU immediately recorded using a multifunctional microplate reader. ATP concentrations were calculated against the standard curve, with high-concentration samples re-measured after dilution. All procedures required light-protected conditions and ice-cold handling. Lysis buffer and detection reagents were stored at -20°C without freeze-thaw cycles. All consumables must be verified ATP-free prior to use.

**Plate Colony Formation Assay**

The plate colony formation assay was employed to evaluate DSHK’s inhibitory effect on the proliferative potential of CRC cells. Logarithmically growing cells were seeded at 800 cells/well in 6 well plates and incubated until attachment. After adherence, the medium was replaced with fresh medium containing gradient concentrations of DSHK for continuous exposure. Following treatment, drug-containing medium was aspirated, and cells were gently washed twice with PBS before replenishing with complete medium for 10-14 days. Fresh medium was supplemented every 3 days to maintain nutrient supply. When macroscopic colonies (diameter ≥0.5 mm) formed, the medium was discarded, and colonies were fixed with 4% paraformaldehyde for 15 min, followed by staining with 0.1% crystal violet solution for 20 min. Colonies containing ≥50 cells were counted under a stereomicroscope to calculate colony formation inhibition rates. Experiments were performed with triplicate technical replicates and repeated in three independent biological replicates.

**EdU Staining Assay**

The 5-ethynyl-2’-deoxyuridine (EdU) labeling method combined with click chemistry was employed to assess DSHK’s impact on CRC cell proliferation. Logarithmically growing cells were seeded at 1×10^4^ cells per well in laser confocal dishes and cultured to 60% confluence in medium containing 10% FBS. The medium was replaced with fresh EdU-labeling medium (10 μM EdU) for 2 h incubation at 37°C to enable DNA incorporation. After medium removal, cells were fixed with 4% paraformaldehyde for 15 min and permeabilized with 0.5% Triton X-100 for 10 min. Click reaction buffer containing Alexa Fluor® 594 azide was added for 30 min light-protected reaction to label proliferating cells. Following DAPI nuclear counterstaining, imaging was performed using a laser confocal microscope (LSM 880, Zeiss). The EdU-positive cell rate (EdU^+^/DAPI^+^ cell ratio) was calculated with ImageJ software, with ≥5 fields analyzed per group across three independent experiments.

**Flow Cytometric Analysis of Apoptosis**

Apoptosis induction by DSHK was evaluated using Annexin V-FITC/PI dual staining combined with flow cytometry. Cells treated with specified DSHK concentrations were digested with 0.25% trypsin (EDTA-free), collected, and washed twice with ice-cold PBS. Cell pellets were resuspended in 100 μL 1× binding buffer, followed by sequential addition of 5 μL Annexin V-FITC and 5 μL propidium iodide (PI, 20 μg/mL). After 20 min of light-protected incubation at 4°C, staining was terminated by adding 400 μL binding buffer, and samples were immediately subjected to flow cytometry. Signal acquisition was performed using FITC (Annexin V) and PE (PI) channels. Apoptotic cell populations were analyzed with FlowJo V10.8 software: Annexin V^+^/PI^-^ defined early apoptosis, Annexin V^+^/PI^+^ represented late apoptosis. ≥10,000 cellular events were recorded per sample, with triplicate independent experiments conducted.

**Immunofluorescence (IF), Immunohistochemistry (IHC), and H&E Staining**

IF: Cell samples were fixed with 4% paraformaldehyde, permeabilized, and blocked, followed by incubation with primary antibodies (1:200, 4°C overnight) and Alexa Fluor® secondary antibodies (1:500) under light-protected conditions. Nuclei were counterstained with DAPI, and images were acquired using a Leica TCS SP8 confocal microscope. Organoid and tissue samples underwent fixation, with organoids embedded in OCT for cryosectioning (8 μm) and tissues paraffin-embedded for sectioning (4 μm). Post-antigen retrieval, staining proceeded identically, with anti-fade mounting medium applied.

IHC: Paraffin sections were dewaxed, rehydrated, and subjected to high-pressure heat retrieval (citrate buffer, 121°C, 3 min). Endogenous peroxidase activity was blocked with 3% H₂O₂. Sections were incubated with primary antibodies (1:100, 4°C overnight), followed by HRP-conjugated secondary antibodies (EnVision™ system) for signal amplification. DAB chromogenic development was performed, nuclei were counterstained with hematoxylin, and sections were mounted with neutral gum.

H&E Staining: Organoids were paraffin-embedded and serially sectioned (4 μm), stained with hematoxylin and eosin, dehydrated, and mounted. Tissue samples were stained with Harris hematoxylin (8 min) and eosin (3 min), followed by gradient dehydration and mounting.

**Molecular Docking**

Small Molecule-Protein Docking: Crystal structures of target proteins were retrieved from the PDB database and preprocessed using AutoDockTools (water removal, hydrogen addition, charge assignment). Small molecule ligands were generated as 3D structures via Open Babel with protonation states corrected for pH 7.4. Docking grids centered on protein active sites were generated by AutoGrid, followed by semi-flexible docking using AutoDock Vina. The lowest binding free energy conformation was selected for interaction analysis. PyMOL visualization depicted proteins as navy blue cartoon models, ligands as cyan stick models, and key binding residues as magenta sticks. Hydrogen bonds (yellow dashed lines), ionic interactions (magenta dashed lines), and hydrophobic contacts (green dashed lines) were explicitly annotated.

Protein-Protein Docking: Receptor and ligand protein structures were predicted by AlphaFold2 and preprocessed with AutoDockTools-1.5.7 (water removal, hydrogen addition, Gasteiger charge assignment). Rigid docking was performed using the GRAMM-X server (collision threshold: -20 kcal/mol, rotational step: 6°), with flexible residue side-chain optimization via FireDock. The global energy-minimized complex conformation was analyzed in PyMOL: receptors as navy blue cartoon models, ligands as cyan cartoon models, interface residues highlighted as colored sticks, and hydrogen-bond networks annotated with yellow dashed lines.

**Molecular Dynamics Simulation Protocols**

Small Molecule-Protein Systems: The molecular dynamics simulations were carried out with Desmond/Maestro noncommercial version 2022.1 as a molecular dynamic’s software. Complex systems were solvated with TIP3P water model and neutralized with 0.15 M NaCl. Following energy minimization, 100 ns NPT ensemble simulations were conducted at 300 K and 1 bar pressure, with trajectories saved every 100 ps. Binding mode stability was analyzed through hydrogen bond occupancy and binding pocket hydration profiles.

Protein-Protein Systems: Simulations were executed using Gromacs 2020. The CHARMM36 force field was applied to proteins, while GAFF2 was used for ligands. Systems were solvated in TIP3P water with sodium/chloride ions added for charge neutralization. Energy minimization employed the steepest descent algorithm, followed by 2000 ps constrained NVT and NPT equilibration to stabilize temperature and pressure. Production MD simulations ran for 100 ns at 300 K, with trajectories saved every 10 ps. Trajectory analysis included root mean square deviation (RMSD), root mean square fluctuation (RMSF), and radius of gyration (Rg).

**Western Blot**

Protein samples were lysed with RIPA buffer containing 1% protease inhibitor cocktail on ice for 30 minutes, followed by centrifugation at 12,000 × g (4°C, 15 min). Supernatants were collected and protein concentrations quantified via BCA assay. Equal amounts of protein (30 μg per lane) were separated by 6-15% SDS-PAGE electrophoresis (stacking gel: 80 V for 30 min; separating gel: 120 V for 90 min). Proteins were transferred to PVDF membranes at 100 V constant voltage for 90 min. Membranes were blocked with 5% non-fat milk in TBST for 1 h at room temperature, then incubated sequentially with primary antibodies (overnight at 4°C with agitation) and HRP-conjugated secondary antibodies (1 h at room temperature). After three TBST washes (10 min each), membranes were incubated with ECL chemiluminescent substrate for 3 min in the dark. Signals were captured using an Amersham Imager 600 system, with band intensities quantified by ImageJ software.

**Cellular Thermal Shift Assay (CETSA)**

The impact of DSHK on target protein thermal stability was assessed using a gradient temperature induction protocol. After 4 h DSHK treatment, equal aliquots of cell lysates (2 mg/mL) were distributed into PCR tubes and incubated at 40°C, 45°C, 50°C, 55°C, 60°C, 65°C, and 70°C for 3 min using a Veriti Pro PCR System (Applied Biosystems). This was followed by room-temperature renaturation for 1 min and immediate transfer to ice to terminate reactions. After centrifugation at 16,000 × g (4°C, 10 min), supernatants were collected for SDS-PAGE electrophoresis (30 μg protein/lane). Target protein retention levels were quantified by Western blotting, with triplicate independent experiments performed per condition.

**Quantitative Real-Time PCR (qRT-PCR)**

Total RNA was extracted using TRIzol reagent, followed by DNase I digestion to eliminate genomic DNA contamination. RNA purity was verified via NanoDrop quantification (A260/A280 ratio = 1.8-2.0). Reverse transcription of 1 μg total RNA was performed with PrimeScript RT Master Mix (Takara) under the following conditions: 42°C for 15 min → 85°C for 5 sec for reaction termination. qPCR reactions (20 μL total volume) contained SYBR Premix Ex Taq II (Takara), 10 μM gene-specific primers (sequences in Table 1), and 2 μL cDNA template. Thermal cycling parameters: initial denaturation at 95°C for 30 sec; 40 cycles of 95°C for 5 sec → 60°C for 30 sec; melt curve analysis (65°C to 95°C with 0.5°C/sec ramp rate). β-actin served as the endogenous control, with relative gene expression calculated using the 2^-ΔΔCt^ method. Each sample was analyzed in triplicate technical replicates across three independent biological experiments.

**Plasmid Construction Methodology**

Full-length human HSPA8 cDNA was PCR-amplified and cloned into the EcoRI/XhoI sites of the pCMV vector to generate a tag-free overexpression plasmid (pCMV-HSPA8). shRNA sequences targeting HSPA8 were inserted into the AgeI/EcoRI sites of the pLKO.1-puro vector for stable knockdown, while synthetic double-stranded siRNA sequences were cloned into the pSI-Check2 vector for transient knockdown. Site-directed mutagenesis was performed using the QuikChange XL kit (Agilent) on the pCMV-HSPA8 template to generate single-point mutants (D10A, T13A, T14A, Y15A) and the quadruple mutant (D10A/T13A/T14A/Y15A). The pGL4.10[luc2] vector was digested with EcoRV/XbaI to excise the luciferase gene, and a multiple cloning site (MCS) was inserted to construct an empty reporter plasmid (pGL4.10-MCS). All plasmids were amplified in E. coli DH5α and validated by restriction enzyme digestion and Sanger sequencing.

**Recombinant HSPA8 Protein Expression and Purification**

The constructed pGEX-4T-1-HSPA8 plasmid was transformed into E. coli BL21(DE3) competent cells. Single colonies were inoculated into 5 mL LB medium (50 μg/mL ampicillin) and cultured at 37°C for 12 h with shaking. Subsequently, 1 mL culture was transferred to 200 mL LB medium and grown at 37°C until OD600 ≈ 0.6. Protein expression was induced with 0.5 mM IPTG at 16°C for 20 h. Cells were harvested by centrifugation (4,000 × g, 10 min, 4°C), resuspended in ice-cold lysis buffer (20 mM Tris-HCl, 500 mM NaCl, 1 mM PMSF, pH 8.0), and lysed by sonication (300 W power, 3s on/5s off cycles, 30 min total). Lysates were centrifuged at 12,000 × g (4°C, 30 min), and supernatants were incubated with pre-equilibrated Glutathione Sepharose^TM^ 4B resin (Cytiva) at 4°C for 2 h. The resin was washed sequentially with 10 column volumes of lysis buffer and 5 column volumes of elution buffer (50 mM Tris-HCl, 10 mM reduced glutathione, pH 8.0). Eluted fractions were treated with PreScission^TM^ protease (GE Healthcare) at 4°C for 12 h to remove the GST tag. Cleaved products were further purified by Superdex^TM^ 75 gel filtration chromatography (GE Healthcare) in 20 mM HEPES, 150 mM NaCl, 0.5 mM TCEP (pH 7.5). Purified protein (>95% purity by SDS-PAGE) was aliquoted and stored at -80°C.

**Pull-Down Assay**

Purified GST-tagged recombinant proteins were incubated with Glutathione Sepharose^TM^ 4B beads in binding buffer (20 mM Tris-HCl, 150 mM NaCl, 0.1% Triton X-100, pH 7.4) at 4°C for 2 h with rotation to construct bait complexes. Cell lysates were prepared using NP-40 lysis buffer (ice-cold lysis followed by centrifugation) and pre-cleared with empty beads for 30 min. Pre-cleared lysates were mixed with bait complexes, supplemented with binding buffer to 500 μL final volume, and co-incubated at 4°C for 4 h with rotation. Beads were sequentially washed five times with 1 mL low-stringency buffer (150 mM NaCl) and five times with high-stringency buffer (500 mM NaCl) to remove non-specific binders. Bound proteins were eluted with 20 mM reduced glutathione (50 mM Tris-HCl, pH 8.0) during 15 min room-temperature rotation. Eluates were resolved by SDS-PAGE and analyzed via Western blotting for target interactors or Coomassie Brilliant Blue staining for interaction profiling. GST-tagged empty vector beads and untransfected cell lysates served as negative controls throughout the procedure.

**Co-Immunoprecipitation (Co-IP)**

Cell lysates were prepared using ice-cold RIPA buffer containing protease inhibitors with 30 min lysis on ice, followed by centrifugation at 12,000 × g (4°C, 15 min) to remove insoluble debris. Supernatants were incubated with specified antibodies on a rotary mixer at 4°C overnight. Pre-equilibrated Protein A/G magnetic beads were added, and incubation continued for 4 h at 4°C with rotation. Bead complexes were washed four times with ice-cold PBS containing 0.1% Tween-20 (1 mL per wash, centrifugation at 1,000 × g, 4°C, 1 min). Bound proteins were eluted by boiling in 1× SDS loading buffer at 95°C for 10 min. Eluates were separated by SDS-PAGE and analyzed via Western blotting for target interacting proteins. Isotype IgG controls and untransfected cell lysates served as negative controls throughout the procedure.

**Cycloheximide (CHX) Chase Assay for Protein Half-Life Determination**

Cells were treated with 100 μg/mL cycloheximide to inhibit nascent protein synthesis. Parallel samples were collected at 0, 4, 8, and 12 h time points, washed twice with ice-cold PBS, and lysed in RIPA buffer containing 1× protease inhibitor cocktail on ice for 30 min. Lysates were centrifuged at 12,000 × g (4°C, 15 min), and supernatants were mixed with 4× SDS loading buffer followed by denaturation at 100°C for 5 min. Target protein levels were analyzed by Western blotting, with band intensities quantified using ImageJ software. β-actin-normalized protein expression values were plotted against time, and protein half-life (t^1/2^) was calculated by fitting the data to a monophasic exponential decay model.

**Plasmid Transfection and Lentiviral Infection Protocols**

Standard Plasmid Transfection: Cells were seeded at 5×10^4^ cells/well in 24 well plates and cultured for 24 h to reach 70% confluence. For each transfection, 1 μg plasmid DNA and 2 μL Lipofectamine 3000 were separately diluted in 50 μL Opti-MEM medium, incubated at room temperature for 5 min, combined, and incubated for an additional 20 min to form liposome-DNA complexes. After removing the original medium, complexes were added dropwise to cells and incubated at 37°C for 6 h before replacing with complete medium. Cells were harvested 48 h post-transfection for analysis.

Lentiviral Packaging and Infection: Packaging plasmids (psPAX2/pMD2.G) and transfer plasmids were co-transfected into HEK293T cells at a 4:3:1 ratio (10 μg total DNA) using the standard transfection protocol. Viral supernatants were collected at 48 h and 72 h post-transfection, filtered through 0.45 μm membranes, and stored at -80°C or used immediately. Target cells were pretreated with polybrene (8 μg/mL) and infected via centrifugation-assisted transduction (1,000 × g, 32°C, 90 min) at MOI = 20. After 24 h incubation at 37°C, medium was replaced with fresh complete medium. Stable transductants were selected using puromycin (1-10 μg/mL) for 48-72 h, with transduction efficiency verified by fluorescence microscopy or flow cytometry.

**Transcriptome Sequencing and Alternative Splicing Analysis**

Total RNA was extracted from cells using TRIzol reagent, treated with DNase I to remove genomic DNA contamination, and subjected to quality control via NanoDrop and Agilent 2100 Bioanalyzer to ensure RNA integrity (RIN ≥8.0). Strand-specific libraries were constructed with the TruSeq Stranded mRNA Library Prep Kit: mRNA was enriched by oligo(dT) beads, fragmented to 350 bp, reverse-transcribed into double-stranded cDNA, end-repaired/A-tailed, and ligated with sequencing adapters followed by 15 cycle PCR amplification. Libraries were quantified by Qubit and qPCR, then sequenced on the Illumina NovaSeq 6000 platform (PE150, ≥6 Gb data per sample). Raw reads were filtered by Trimmomatic to remove low-quality sequences, aligned to the GRCh38 reference genome using HISAT2, and assembled into transcripts via StringTie. Alternative splicing (AS) events were analyzed using rMATS (v4.1.2) to detect seven splicing types (e.g., skipped exons, alternative 5’/3’ splice sites), with significant events defined as FDR <0.05 and |ΔPSI| ≥0.1. Key splicing isoforms of target genes were validated by RT-qPCR using primers spanning splice junctions.

**Bioinformatics and Conservation Analysis**

Survival curves were constructed based on the Kaplan-Meier method. STAR-Counts data and clinical information for CRC were obtained from the TCGA database (https://portal.gdc.cancer.gov). TPM values were log2(TPM+1) normalized, and 1,609 samples with matched RNA-seq and clinical data were selected for downstream analysis. Single-sample gene set enrichment analysis (ssGSEA) was performed using the GSVA package (R v4.0.3, method = “ssgsea”) based on the KEGG CRC pathway gene set (hsa05210). Spearman correlation tests assessed associations between target genes and pathway activity. For survival analysis: patients were stratified into high/low expression groups by median target gene expression. Kaplan-Meier survival curves with log-rank tests (P<0.05 significance threshold) evaluated intergroup differences, while univariate Cox regression calculated hazard ratios (HR) and 95% confidence intervals (CI). Amino acid conservation was systematically evaluated using PolyPhen-2 (missense variant pathogenicity prediction), PROVEAN (functional impact scoring), PoPMuSiC (protein stability change prediction), and VarSite (structural conservation analysis) to determine evolutionary conservation of key residues (e.g., ASP10) across vertebrate species.

**Human CRC Tissue Microarray (TMA)**

A commercial human CRC tissue microarray (80 paired tumor/adjacent tissues, core diameter: 1.5 mm; Hunan Aifang Biotechnology Co., Ltd.) was utilized. TMAs were rehydrated by 30 min incubation at 60°C, followed by xylene dewaxing (15 min ×2) and gradient ethanol hydration (100%→80%). Antigen retrieval was performed with EDTA buffer (pH 9.0) via microwave heating (800 W, 8 min). Endogenous peroxidase activity was blocked with 3% H_2_O_2_ (room temperature, 15 min). Primary antibodies (1:200 dilution) were incubated overnight at 4°C in a humidified chamber, followed by HRP-conjugated secondary antibodies (1 h, room temperature). DAB chromogenic development (monitored microscopically for 30-90 sec) and hematoxylin counterstaining (1 min) were performed. Each batch included positive controls (known expression tissues) and negative controls (PBS instead of primary antibody). Whole slides were digitally scanned (Aperio AT2 scanner), with H-scores (0-300) assessed by two independent pathologists blinded to sample groups.

**Supplementary tables for methods**

**Table 1. Antibody Information**

| **Antibodies** | **Source** | **Catalog Number** |
| --- | --- | --- |
| Anti-FASN | Cell Signaling Technology | 3180S |
| Anti-PLEC | Cell Signaling Technology | 4395S |
| Anti-MYH9 | Cell Signaling Technology | 3403S |
| Anti-IQGAP1 | Cell Signaling Technology | 3278S |
| Anti-HSP90 | Cell Signaling Technology | 4877S |
| Anti-PRPF8 | Cell Signaling Technology | 3471S |
| Anti-PKM | MedChemExpress | HY-P80870 |
| Anti-HSPA5 | MedChemExpress | HY-P84053 |
| Anti-HSPA8 | MedChemExpress | HY-P80708 |
| Anti-NQO1 | ABclonal | A11494 |
| Anti-NQO2 | ABclonal | A11294 |
| Anti-β-catenin | Proteintech | 51067-2-AP |
| Anti-U2AF2 | Proteintech | 20140-1-AP |
| Anti-GEMIN5 | Proteintech | 20141-1-AP |
| Anti-GST | Abcam | ab19256 |
| Anti-HSPD1 | Abcam | ab3080 |
| Anti-HA | Abcam | ab49969 |
| Anti-p-eIF2α | Abcam | ab32157 |
| Anti-eIF4A | Abcam | ab31217 |
| Anti-eIF4E | Abcam | ab81242 |
| Anti-eIF4G | Abcam | ab2605 |
| Anti-RPL11 | Selleck | F1403 |
| Anti-RPL8 | Selleck | F3288 |
| Anti-RPL35 | Santa Cruz | sc-390992 |
| Anti-RPL3 | Santa Cruz | sc-134994 |
| Anti-RPS14 | Santa Cruz | sc-390992 |
| Anti-Puromycin | Merck | MABE343 |
| Anti-β-actin | Cell Signaling Technology | 3700S |
| Secondary antibodies | Cell Signaling Technology | 7076P2 or 7074P2 |
|  |  |  |

**Table 2. Primer pairs for qRT-PCR**

| **Gene** | **Sequence** |
| --- | --- |
| ***Hspa8*** | **F**: 5’-ACTCCAAGCTATGTCGCCTTT-3’  **R:** 5’-TGGCATCAAAAACTGTGTTGGT-3’ |
| ***β-actin*** | **F:** 5’-GGCACCCAGCACAATGAA-3’  **R:** 5’-GCTGATCCACATCTGCTGG-3’ |
| ***rRNA（ITS1）*** | **F**: 5’-CGACCCGGGGAGGTAGTG-3’  **R:** 5’-TGCGCTGCCTTGGTACCG-3’ |
| ***18S*** | **F**: 5’-GTAACCCGTTGAACCCCATT-3’  **R:** 5’-CCATCCAATCGGTAGTAGCG-3’ |
| ***RPS14*** | **F**: 5’-TGGAGACGACGTGCAGAAAT-3’  **R:** 5’-TGGCAGACACCAAATACATTCT-3’ |
| ***RPL35*** | **F**: 5’-CTCTAAGATCCGAGTCGTCCG-3’  **R:** 5’-GCTTGTACTTCTTGCCCTTGTAG-3’ |
| ***RPL11*** | **F**: 5’-TCCCGTTTCTATCCAAAAGAGCA-3’  **R:** 5’-CCAGCTACTCCCATCGAAGC-3’ |
| ***RPL3*** | **F**: 5’-ATTGCCCACACCCAGATGC-3’  **R:** 5’-CCCAAACACTTGGTTCACAGG-3’ |
| ***RPL8*** | **F**: 5’-GACAGCCAGAGCCGACAT-3’  **R:** 5’-CTGTCATGTGCCGGCATTA-3’ |

**Table 3. The sequences of siRNAs**

| **siHSPA8 1#** | 5’-3’ (sense) GUCCUCAUCAAGCGUAAUATT  5’-3’ (antisense) UAUUACGCUUGAUGAGGACTT |
| --- | --- |
| **siHSPA8 2#** | 5’-3’ (sense) GGCCAGUAUUGAGAUCGAUTT  5’-3’ (antisense) AUCGAUCUCAAUACUGGCCTT |
| **N.C.** | 5’-3’ (sense) UUCUCCGAACGUGUCACGUTT  5’-3’ (antisense) ACGUGACACGUUCGGAGAATT |

**Table 4. Lentiviral Sequences Targeting HSPA8 Gene for Interference**

| **Name** | **Target Gene** | **Sequence (5’-3’)** |
| --- | --- | --- |
| HSPA8 1# | HSPA8-Homo-1790 | TGGACAAGTGTAATGAAATTA |
| HSPA8 2# | HSPA8-Homo-212 | CGGACACTGAACGGTTGATCG |
| HSPA8 3# | HSPA8-Homo-743 | CAGCTGGAGACACCCACTTGG |
| HSPA8 4# | HSPA8-Homo-973 | CGTGCCCGATTTGAAGAACTG |
| LV-3NC |  | TTCTCCGAACGTGTCACGT |
